# Extensive Band Gap Tunability in Covalent Organic Frameworks via Metal Intercalation and High Pressure

**DOI:** 10.1021/acs.jpclett.5c01216

**Published:** 2025-07-15

**Authors:** Michelle Ernst, Jürg Hutter, Stefano Battaglia

**Affiliations:** Department of Chemistry, 27217 University of Zurich, 8057 Zürich, Switzerland

## Abstract

Covalent organic frameworks (COFs) are materials of growing interest for electronic applications due to their tunable structures, chemical stability, and layered architectures that support extended π-systems and directional charge transport. While their electronic properties are strongly influenced by the choice of molecular building blocks and the stacking arrangement, experimental control over these features remains limited, and the number of well-characterized COFs is still relatively small. Here, we explore two alternative strategies, hydrostatic pressure and metal intercalation, to tune the electronic structure of COFs. Using periodic density functional theory (DFT) calculations, we show that the band gap of pristine COF-1 decreases by ∼1 eV under compression up to 10 GPa. Metal intercalation induces an even greater reduction, in some cases leading to metallic behavior. We demonstrate that pressure and intercalation offer effective, continuous control over COF electronic properties, providing powerful means to complement and extend conventional design approaches.

## Graphical abstract

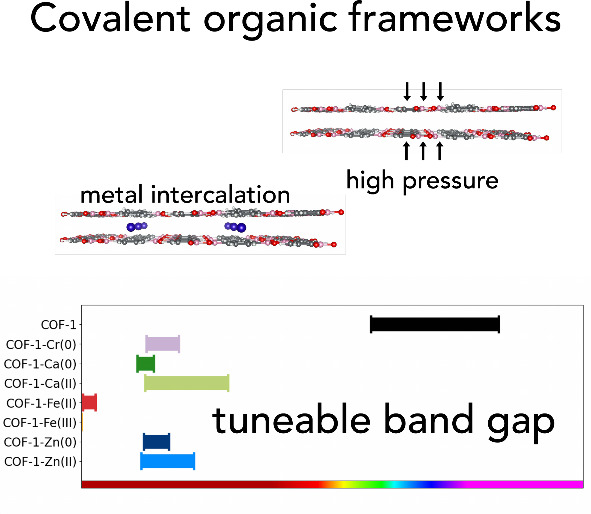

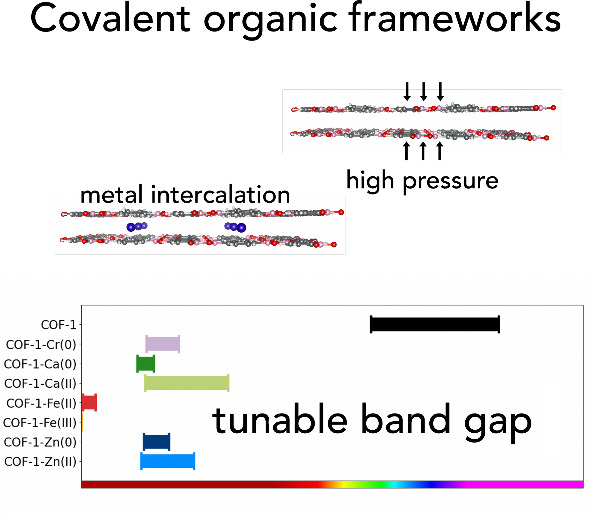

Covalent organic frameworks (COFs) are a class of crystalline, porous materials constructed from organic building blocks connected via strong covalent bonds. Their ordered structures, large internal surface areas, and tunable porosity make them promising for applications in gas storage and separation, drug delivery, catalysis, and optoelectronics.^1^ In particular, COFs possess several characteristics that make them attractive for electronic applications: they are lightweight, mechanically and thermally robust, and composed of earth-abundant elements, offering an environmentally sustainable alternative to conventional charge-transport materials in electronic devices. However, most COFs exhibit band gaps in the range of 2–4 eV,^2^ classifying them as semiconductors or insulators. This range is suitable for some optoelectronic applications, though, it restricts their use in devices requiring metallic conductivity or extremely wide band gaps. Expanding the accessible band gap range, particularly below 2 eV, is essential, as many electronic applications demand lower band gaps and precise control over their tuning.^3–5^ For instance, photocatalytic CO_2_ reduction and hydrogen evolution require materials with band gaps in the 1.5–2.0 eV range to efficiently absorb visible light while maintaining redox activity.^3^ Organic field-effect transistors benefit from narrow band gaps to reduce charge injection barriers and enable ambipolar transport.^6^ Narrower band gaps are also essential in flexible straintronic devices, where even small deformations must induce measurable electronic changes.^4^ More broadly, band gap engineering is a central design principle across technologies ranging from transistors to light harvesting systems.^7^ Enabling precise and continuous control over the electronic properties of COFs would significantly broaden their functional scope and facilitate their integration into next-generation electronic materials.

Several strategies have been explored to tune the electronic properties of COFs, such as modifying linkers and building blocks,^8–10^ altering their topology,^11^ introducing dopants,^12,13^ incorporating metals,^14–16^ or tuning interlayer stacking motifs.^17^ These approaches have demonstrated some success in tuning COF band gaps, charge carrier densities, and charge transport properties. However, the number of experimentally realized COFs remains limited, as reflected in databases such as CoRE-COF-v7.0 (1242 structures).^18^ A major challenge lies in the difficulty of synthesizing well-ordered single crystals, complicating structure determination, especially with respect to their stacking arrangements.^19^ This structural uncertainty makes it difficult to systematically control and optimize the electronic properties of COFs, limiting their practical implementation.

To overcome these limitations, alternative tuning strategies are necessary. In this work, we computationally explore two such approaches: metal intercalation and high pressure, as well as their combined effects. Intercalation involves the insertion of metal atoms or molecules into a host material, fundamentally altering its electronic, catalytic, and structural properties.

Metal intercalation in COFs was first proposed in theoretical studies.^20^ Subsequent computational investigations demonstrated that intercalation can significantly reduce the band gap, transforming COFs into conductors.^21–25^ By selecting specific metals and intercalation sites, electronic properties such as the band gap and density of states can be precisely tuned, expanding the potential of COFs for electronic applications. Beyond their role as semiconductors, intercalated COFs have been proposed for spintronic and magnetic applications,^25^ as well as in photocatalysis^26,27^ for oxygen evolution and hydrogen reduction reactions.^25^ Their potential in energy storage has also been explored.^20,28^ Although intercalation has been predominantly studied computationally, recent reports have demonstrated its experimental realization.^27,28^

The application of high pressure offers another promising strategy for modifying the electronic properties of COFs. In contrast to temperature, which often leads to thermal degradation at elevated levels, pressure can induce structural rearrangements and modify the electronic structure without altering chemical composition. It has been shown to transform numerous materials from insulators into semiconductors or conductors.^29^ However, its potential for COF band gap modulation remains unexplored. In contrast to intercalation, which introduces discrete chemical modifications, high pressure provides a continuous means of tuning electronic properties by altering bond lengths, stacking arrangements, and electronic band structures. As a result, pressure enables regions of the free-energy landscape to be reached that would otherwise be inaccessible, making it a powerful tool for material design. To date, only a few studies have systematically examined the effects of high pressure on COFs experimentally,^30,31^ and computationally,^32^ highlighting the need for further exploration. Beyond fundamental insights, high-pressure studies that focus on both structure and electronic structure have practical applications, including the design of stress-resistant flexible electronics, pressure-sensitive electronic switches, and high-performance pressure sensors.^33,34^ COFs, with their intrinsically low bulk modulus, are particularly responsive to pressure, yet structurally robust, making them promising candidates for such applications.

In this study, we present the first computational investigation of high-pressure effects on COF band gaps and the first combined analysis of high pressure and intercalation as tuning mechanisms. We demonstrate that band gaps of COFs are highly tunable through metal intercalation and high pressure, making them suitable for a wider range of electronic applications from semiconductors to conductors. Thus, our work provides a new way for tailoring COF electronic properties and thus their integration into next-generation functional materials.

To investigate these effects, we selected COF-1, a prototypical two-dimensional (2D) COF. A staggered stacking arrangement with a layer offset of approximately 1.4 Å was adopted as starting structure, as this configuration has been identified as the most stable by Lukose et al.^35^ Four metal species were systematically intercalated between benzene rings in a ratio of one metal per unit cell. Ca and Zn were chosen as they represent the two extremes among the first-row transition metals, while Cr and Fe occupy intermediate positions. Ca was specifically included as it was also part of the study of Gao et al.^20^ Cr(0) was chosen due to the well-established stability of bis­(benzene)chromium complexes, where it is also intercalated between benzene rings.^36^

We performed unrestricted Kohn–Sham density functional theory (DFT) calculations using the r^2^SCAN functional^37^ and the Gaussian and plane wave approach,^38^ in combination with the DZVP-MOLOPT-SR-GTH basis set^39^ and the fully nonlocal Goedecker-Teter-Hutter pseudopotential.^40^ A comparison of r^2^SCAN to other commonly used functionals is provided in Section 1.2 of the Supporting Information (SI).

A grid cutoff of 700 Ry and a relative cutoff of 60 Ry were used for the multigrid setup containing 4 grids. The Brillouin zone was sampled using a Monkhorst–Pack *k*-point grid of 3×3×7, which was found to provide converged energies (cf. Table S1). The electronic properties of all structures were analyzed by computing the density of states (DOS) and band structures along the high-symmetry path Γ–M–K−Γ–A–L–H–A–M–H in the first Brillouin zone. Crystalline orbitals and projected DOS (pDOS) were calculated at the Γ point. All calculations were performed in CP2K version 2024.3.^41^

One unit cell consists of two layers in the c-direction and a total of 84 atoms (85 for intercalated systems) and was constrained to be hexagonal. The atomic positions and cell parameters of all systems were optimized using the conjugate gradients optimizer until convergence, and a subsequent vibrational analysis for all structures at ambient pressure resulted in no imaginary frequencies.

To assess the effect of compression on the electronic structure, external pressures of 2.5 and 5 GPa were applied. In addition, pristine COF-1 was studied under 7.5 and 10 GPa of hydrostatic pressure. The bulk modulus was determined by fitting the third-order Birch–Murnaghan equation of state to the calculated pressure–volume data.

The chosen intercalated metals, along with their formal oxidation states and lowest-energy spin states, are summarized in Table S6. For systems with ambiguous spin states (Cr, Fe), geometry optimizations with different multiplicities were performed to identify the most stable multiplicity. In all cases, the low spin state was the most stable (cf. Table S5). For Cr, this is in agreement with DFT calculations on the bis­(benzene)chromium complex.^36^

Additional information on the computational details are given in Section 1 of the SI.

We start by presenting the structural response of COF-1 under pressures of up to 10 GPa. The evolution of the unit cell parameters up to 5 GPa is shown in Figure 1a, while Table S7 provides the complete set of computed structural parameters, including unit cell volumes. Table S8 provides information on the porosity (surface area, helium volume, density) of all systems. The lattice parameters *a* and *b* (the same due to hexagonal symmetry) exhibit a slight decrease with increasing pressure, whereas the *c*-axis contracts significantly, with a reduction of approximately 1 Å already at 5 GPa. Our computational results align well with the high-pressure experimental data reported by Sun et al.^30^ (cf. Figure S1), which remains the only experimental study of COFs under compression.

**Figure 1:**
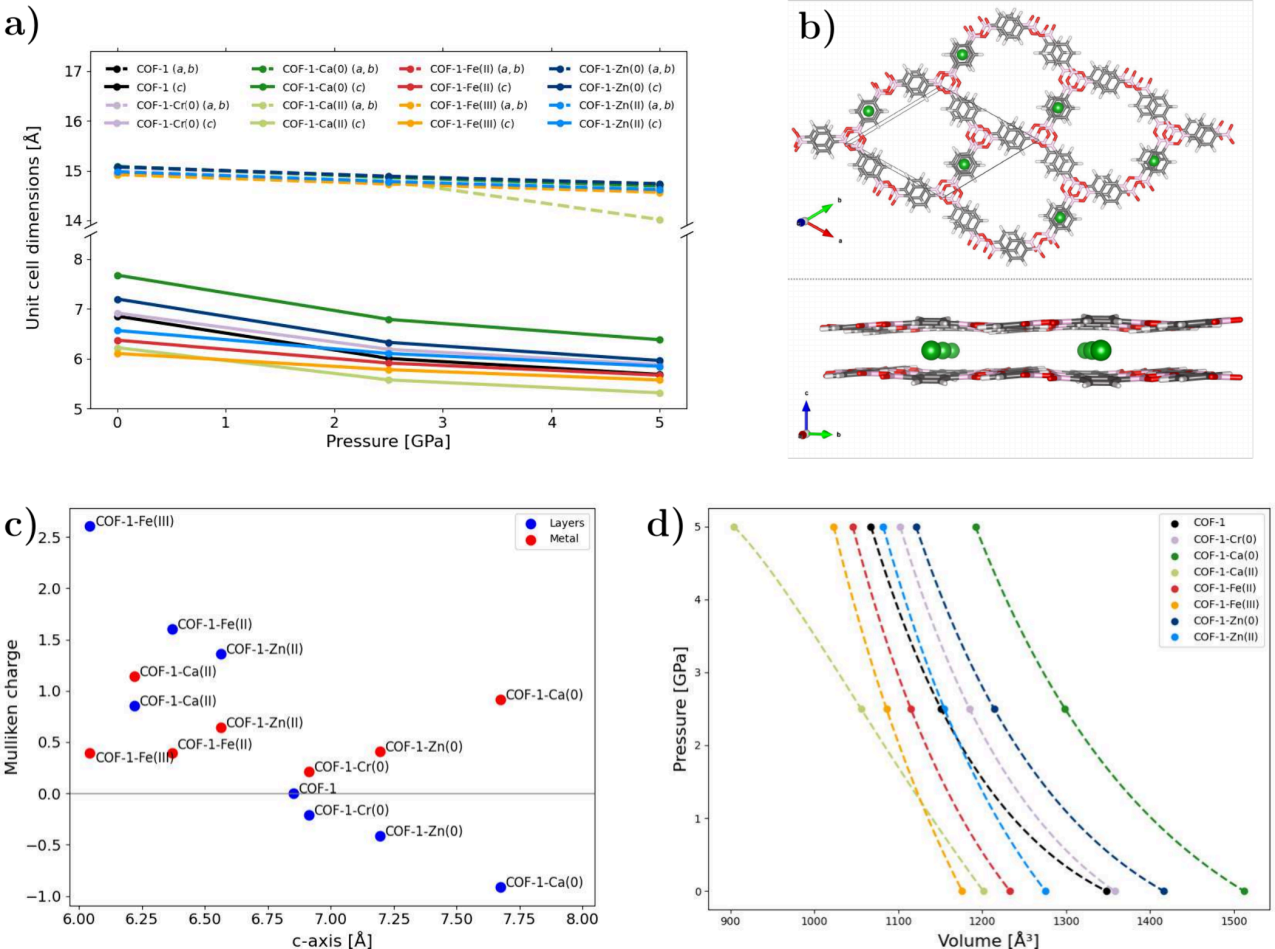
(a) Optimized unit cell dimensions *a*, *b*, *c* of the hexagonal COF-1-M crystals at 0, 2.5, and 5 GPa. (b) Structural representation of COF-1-Ca(0), illustrating an intercalated COF-1. (c) Mulliken charges on the framework (blue) and on the metal atom itself (red) as a function of the unit cell *c*-axis length. (d) Birch–Murnaghan fits for bulk moduli.

Figure 1a also illustrates the optimized unit cell parameters (*a*, *b*, *c*) for COF-1 structures intercalated with metal species (COF-1-M) (cf. Figure 1b for an exemplary structure). At ambient pressure, Ca(0), which has the largest van der Waals radius among the selected metals, induces the most significant expansion of the *c*-axis and interlayer spacing (cf. Figures 1a and S2).

As illustrative examples, we verified the stability of Ca(0), Ca­(II), and Fe­(II) intercalation and identified the adsorption site between the benzene rings as the most favorable. In case of Ca(0), the binding energy is −248.7 kJ/mol, in good agreement with the result reported by Gao et al.,^20^ who obtained a slightly lower binding energy in a system with higher metal loading and a different computational setup. More information is provided in Section 2.4 of the SI. Intercalation of Cr(0) and Zn(0) lead to only minor increases of the *c*-axis. In contrast, the insertion of Ca­(II), Fe­(II), Fe­(III), and Zn­(II), that is, all charged metal species, leads to a contraction of the *c*-axis. This effect is likely driven by electrostatic interactions. Mulliken charge analysis indicates that the positive charge is not fully localized on the metal but is redistributed over the COF layers (cf. Figure 1c). In neutral intercalated systems, the total charge remains the same as in pristine COF-1. The Mulliken analysis (cf. Figure 1c) shows an accumulation of positive charge on the metal, which is compensated for by a more negatively charged framework. This increased electron density on the framework enhances the interlayer repulsion and leads to an expansion of the *c*-axis. Larger partial charges correspond to larger *c*-axes.

On the other hand, in charged systems, the framework has a net positive charge and, consequently, a reduced electron density above and below the layers. This decreases the electrostatic repulsion between them and results in a contraction of the *c*-axis. A special case is COF-1-Ca­(II), where the layers shift laterally, altering the stacking geometry and reducing interlayer repulsion.

Interestingly, the Mulliken charges on the metal centers differ only slightly between neutral and charged systems of the same element suggesting that the overall framework charge distribution, rather than the formal metal oxidation state, or electrostatic attraction between the intercalated ions and the framework layers, plays the dominant role in determining the structural response.

Compression of the COF-1­(-M) structure leads to a relatively small reduction in the *a* and *b* unit cell dimensions (from approximately 15 Å to 14.5 Å), as expected for the covalently bonded in-plane directions. In contrast, the *c* lattice parameter decreases more, highlighting the weaker van der Waals interactions between the layers. While pristine COF-1 remains completely flat up to 10 GPa, metal intercalation introduces a slight distortion of the layers. Among the studied species, Ca­(II) undergoes the most pronounced structural rearrangement: already at ambient pressure, the layers undergo a lateral shift and slip along the *ab* plane, such that some boroxine rings in one layer align with the benzene ring in the other layer in contrast to the offset stacking observed for all the other systems considered (cf. Figure S3). In this arrangement, there is a much stronger interaction between an oxygen atom of the boroxine ring and the calcium ion. Upon compression to 5 GPa, the layers are no longer fully planar, and the *a* and *b* lattice parameters contract more strongly than in the other intercalated systems, consistent with the altered stacking geometry (cf. Figure 1a).

The bulk modulus of pristine COF-1 is 7.8 GPa. Upon metal intercalation, the bulk modulus varies depending on the metal species—Ca(0): 10.6 GPa, Ca­(II): 18.7 GPa, Cr(0): 10.8 GPa, Fe­(II): 18.0 GPa, Fe­(III): 26.2 GPa, Zn(0): 9.3 GPa, and Zn­(II): 18.9 GPa. The corresponding fits are presented in Figure 1d. Our results indicate that COF-1-Fe­(III) is the stiffest among the systems studied, while the pristine COF-1 is the most compressible. All intercalated systems exhibit relatively low bulk moduli compared to other two-dimensional materials, such as MoS_2_ (79.5 GPa),^42^ black phosphorus (34 GPa),^43^ and graphite (30.8 GPa experimental,^44^ ∼30 GPa theoretical^45^). 2D hybrid perovskites have comparable bulk moduli, such as for example (BA)_2_PbBr_4_ (BA = benzylammonium) (10 GPa)^46^ and BA_2_MAPb_2_I_7_ (BA = benzylammonium, MA = methylammonium) (12.3 GPa).^47^

Having analyzed the structural response of COF-1 and its metal-intercalated derivatives under pressure, we now turn to the corresponding electronic properties. The band gap of COF-1 decreases with increasing pressure, from 3.33 eV at 0 GPa to 2.86 eV at 5 GPa and further to 2.31 eV at 10 GPa (cf. Figure 2a and Table S10). Thus, in this experimentally accessible pressure range, a significant reduction of 1.0 eV is achieved. The electronic structure of COF-1 exhibits a relatively flat band dispersion (Figure 2c, left), though not entirely dispersionless. At higher pressures, the shorter distance between the layers increases their interaction and orbital overlap (Figure 2b), resulting in a larger band dispersion (cf. Figures S5–S7).

**Figure 2:**
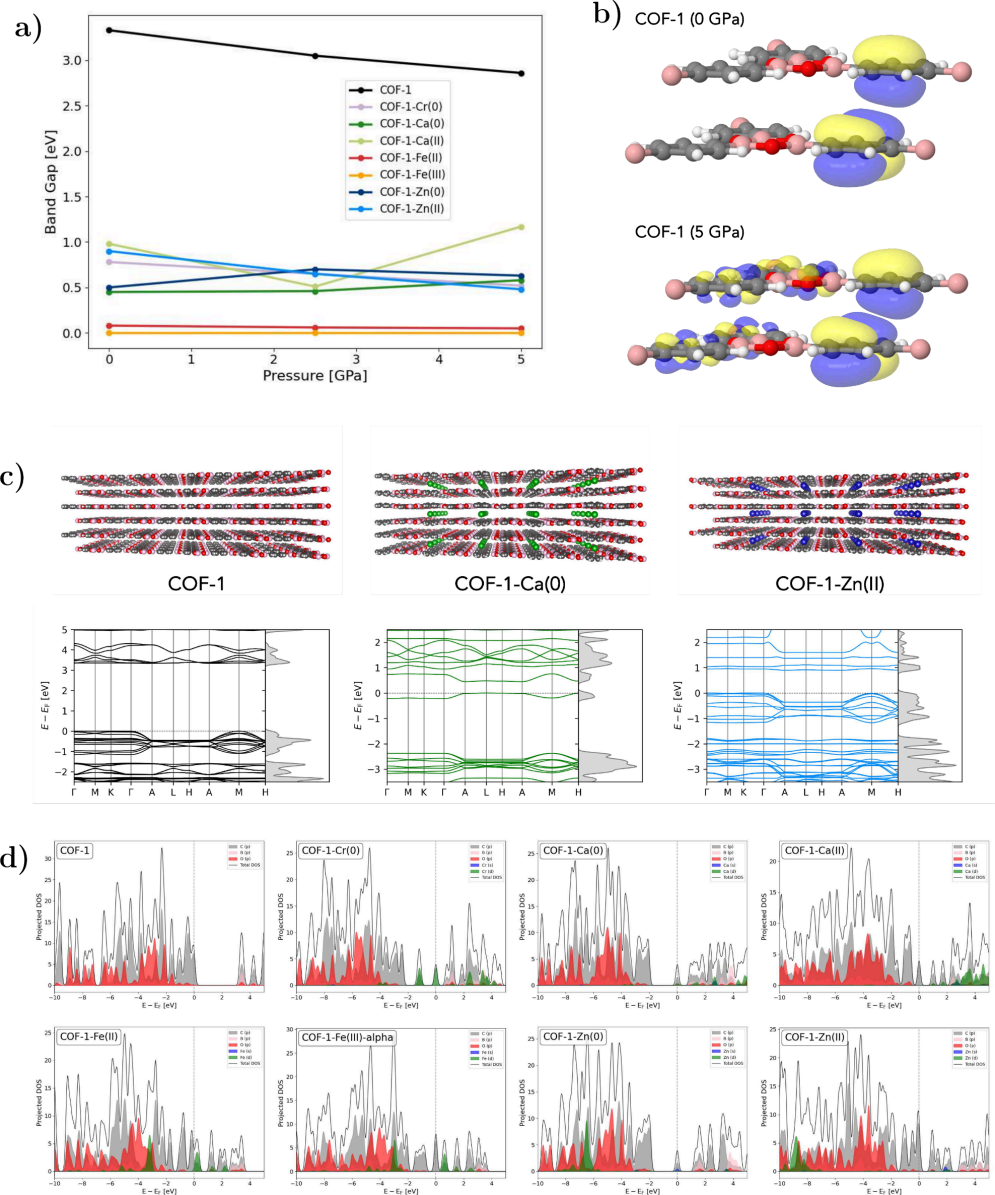
(a) Band gaps of COF-1­(-M) at 0, 2.5, and 5 GPa. (b) HOCO at the Γ point of the pristine COF-1 at 0 and 5 GPa with a contour level of 0.03. (c) Band structures and DOS of the pristine COF-1, COF-1-Ca(0), and COF-1-Zn­(II) at 0 GPa. (d) Projected density of states at the Γ-point for COF-1-M structures at 0 GPa.

In all cases, metal incorporation leads to a substantial reduction in the band gap, bringing the system close to a conductive state. Nevertheless, the characteristic band pattern of COF-1 remains visible in all cases (cf. Figure S5). The following discussion focuses first on the electronic structures at 0 GPa.

Ca­(II), having no electrons in the 4s or 3d orbitals, contributes only through lower-lying 3s and 3p states to occupied bands. As a result, the highest occupied bands are dominated by states from the COF framework, as illustrated by the projected density of states (pDOS) in Figure 2d. However, Ca­(II) does contribute to the unoccupied bands just above the Fermi energy. Ca(0) introduces two additional electrons giving rise to an additional filled band. This band lies significantly above the next lower-lying occupied states (Figure 2c, center). A Γ-point calculation shows that this highest occupied band is primarily composed of carbon p-orbitals and calcium d-orbitals, as evident from the pDOS (Figure 2d) and the visualization of the highest occupied valence band or highest occupied crystalline orbital (HOCO) at the Γ-point (cf. Figure 3). COF-1-Ca(0) displays one of the lowest band gaps of 0.45 eV even though it has the largest *c*-axis.

**Figure 3:**
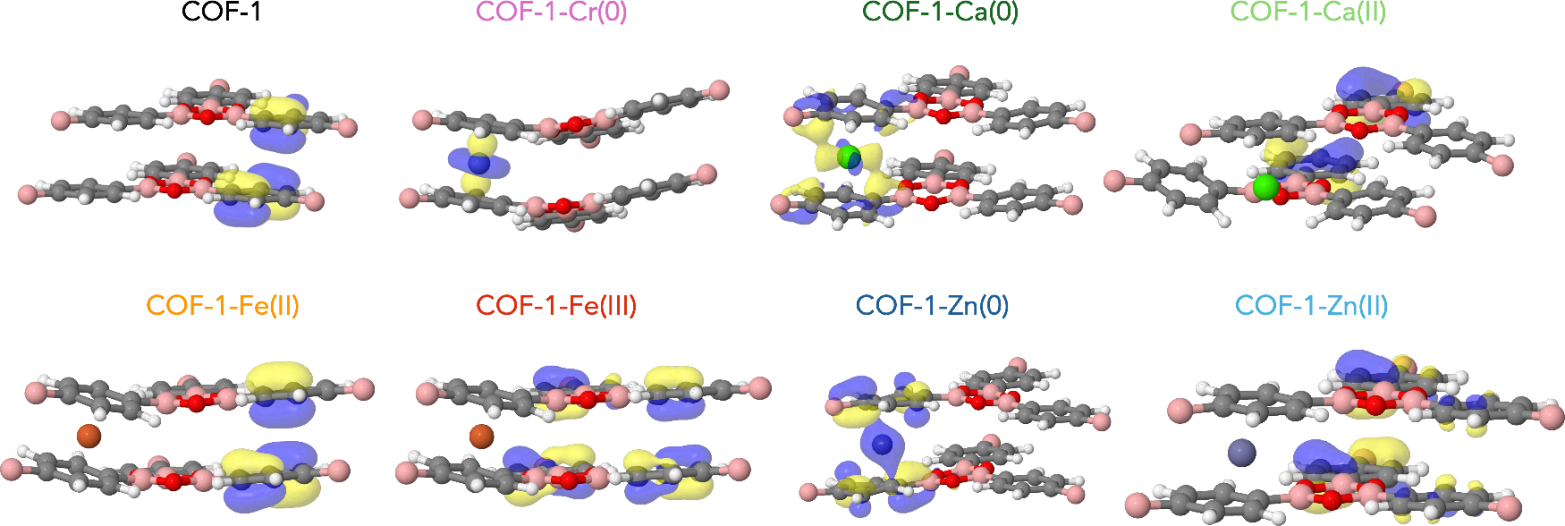
HOCOs from Γ-point calculations at 0 GPa.

We ascribe this to the partially occupied 3d states, which are energetically close to each other. Only Fe­(II) and Fe­(III) induce a more pronounced reduction of the band gap, leading to a metallic character. COF-1-Zn(0) has a band structure similar to that of COF-1-Ca(0). The highest occupied band is likewise dominated by carbon p-orbitals and zinc states, though in this case the main contribution originates from the Zn 4s orbital, while the fully occupied 3d orbitals lie at lower energy (cf. Figure 2d). In Zn­(II), this band is no longer occupied, but the remaining bands shift significantly closer to the conduction band (Figure 2c, right). Additionally, the band dispersion is increased in Zn­(II) compared to the corresponding bands in Zn(0), which may be attributed to the reduced interlayer spacing in Zn­(II) and increased electronic overlap between the COF layers.

Among the intercalated COFs, Ca­(II) exhibits the largest band gap at ambient pressure, followed closely by Zn­(II). In both cases, the highest occupied valence band of the Γ-point calculation is not localized on the metal, suggesting that metal states do not directly contribute to the highest occupied band. However, the lowest conduction band does contain metal contributions, as shown by the pDOS in Figure 2d.

Cr­(0), with a partially filled 3d shell, falls between these cases. The HOCO of COF-1-Cr(0) is a well-defined 3d orbital, as evident from the pDOS (Figures S5–S7) and HOCO (Figure 3). For Fe­(II) and Fe­(III), the occupied d-states are located at lower energies, while the unoccupied d-states contribute to the lowest conduction bands.

All band gaps are indirect, though in some cases, as a consequence of the nearly flat bands, the direct band gaps are of comparable magnitude.

Applying pressure on the COF-1-M further extends the tunable range of their electronic properties, enabling a broader adjustment of conductivity. For Ca­(II), Cr(0), Fe­(II), and Zn­(II), the band gap decreases with increasing pressure, consistent with enhanced interlayer orbital overlap due to reduced layer spacing. An exception is observed for Ca­(II) at 5 GPa, where a structural rearrangement alters the electronic structure and reverses the expected trend.

Two additional deviations from this trend are observed: for COF-1-Zn(0), the band gap slightly increases between 0 and 2.5 GPa, and for COF-1-Ca(0), an increase is seen from 2.5 to 5 GPa, even though the interlayer spacing decreases and no major structural changes are detected. In both cases, the HOCO shows a substantial contribution from the metal atoms (see Figure 3), unlike in COF-1-(Ca­(II), Fe­(II), Fe­(III), Zn­(II)) where the frontier orbitals are primarily on the organic framework. This suggests that when the highest occupied band is dominated by the metal, the relationship between interlayer spacing and band gap becomes less straightforward. Cr(0) appears to be an exception: although its HOCO is also metal-centered, it still follows the general trend of decreasing band gap with reduced spacing. A comparable phenomenon was observed by Ling and Slater for the breathing MOF MIL-53­(M) (M = trivalent metal), where the band gap in the narrow-pore form was consistently smaller than in the large-pore form across several metal variants.^48^ In MIL-53-Al, where the HOCO and LUCO are primarily located on the organic linker, the reduced band gap in the narrow-pore form was attributed to enhanced orbital overlap from closer proximity of benzene rings, an interpretation that parallels our observations. However, in MIL-53 variants with transition-metal cations, where metal d-states contribute significantly to the HOCO, the correlation between pore size and band gap weakened. This again mirrors our observations for COF-1-Ca(0) and COF-1-Zn(0), in which the HOCO is centered on the intercalated metal rather than the organic COF layer, and the expected relationship between reduced interlayer spacing and band gap narrowing no longer strictly holds. These system-specific deviations reflect the complex interplay of structural and electronic effects under pressure. Nonetheless, the overall trend confirms that pressure is a continuous tuning parameter with a significant influence on the electronic structure of COFs.

In summary, the behavior of pristine COF-1 follows a clear trend, where increasing pressure leads to a reduction in interlayer spacing and a progressively smaller band gap. This behavior is consistent with trends observed in a range of layered two-dimensional materials, including perovskites,^49–52^ transition-metal dichalcogenides,^53,54^ transition-metal dihalides,^55,56^ transition-metal phosphorus trisulfides,^57^ black phosphorus,^58^ and antimony,^59^ where compression narrows the band gap (cf. Section 5 in the SI for an extended discussion). A notable exception is graphene, a gapless material at ambient pressure, which exhibits a band gap opening under pressure.^60^ The band gap reduction observed in COF-1 under pressure exceeds that reported for most of the aforementioned materials, indicating a particularly strong electronic response to compression relative to other layered two-dimensional systems. Metal–organic frameworks also offer broad opportunities for band gap tuning. For an overview of the electronic properties of 2D MOFs and strategies to tune them, we refer the reader to the recent review by Lu et al.^61^

In our study, metal intercalation, irrespective of the specific metal species or its oxidation state, consistently leads to a significant band gap reduction compared to the pristine COF. This result aligns with broader trends reported in the literature, where intercalation is widely recognized as an effective strategy for tuning the electronic properties of layered materials.^7,62–64^

We identified two distinct mechanisms by which metal intercalation alters the electronic structure of COF-1. On the one hand, intercalants introduce metal-derived bands within the original band gap, effectively reducing the gap by creating new electronic states between the valence and conduction bands. On the other hand, they modify the electronic structure through shifts in the Fermi level and changes in charge distribution. This is consistent with observations in other two-dimensional systems, where even low levels of intercalation, comparable to doping, are highly effective in tuning interlayer interactions and electronic properties.

When pressure is applied to metal-intercalated COFs, the general trend of decreasing interlayer spacing and narrowing band gap remains valid in most cases. However, certain systems deviate from this pattern. In COF-1-Zn(0) and COF-1-Ca(0), the band gap does not continuously decrease with pressure, indicating that the combined influence of geometric and electronic factors on both, metal and layer, can lead to responses that are not straightforward to interpret. In the more pronounced case of COF-1-Ca­(II), pressure induces structural rearrangements that disrupt the planarity of the layers, causing its behavior to diverge more markedly from the other systems. These observations underscore the sensitivity of electronic properties to geometric factors such as planarity and stacking arrangement, which remain challenging to precisely resolve experimentally,^65,66^ and they highlight the importance of computational investigations that can provide detailed insight into structure–property relationships often inaccessible by experiment alone.

Altogether, our results show that pressure and metal intercalation are effective and complementary tools for modulating the electronic properties of COFs. Their combination enables access to a wider range of electronic behaviors than either approach alone. The interplay between structural changes and electronic response is nontrivial and requires detailed analysis, as demonstrated here. Further experimental studies will be essential to validate these predictions and to clarify how external stimuli shape the structure and function of COFs in practice.

## Supplementary Material





## Data Availability

All computation files are openly available in the Materials Cloud Archive 2025.63 (2025), 10.24435/materialscloud:yw-3f.

## Notes

Computational details and parameter selection (cell optimization COF-1 at 0 GPa, exchange-correlation functionals comparison, charge and spin states); Geometric features of intercalated COFs (unit cell parameters, surface area and density, comparison of cell parameters to experiment, binding energies and adsorption sites, interlayer distance, structural rearrangement COF-1-Ca­(II)); Electronic structure; Triazine-based COF-IITI-0; Pressure dependence of the band gap in other layered two-dimensional materials (PDF)
Transparent Peer Review report available (PDF)

## ‡

Institute of Geological Sciences, University of Bern, 3012 Bern, Switzerland

## References

[1] Ding S. Y., Wang W. (2013). Covalent organic frameworks (COFs): From design to applications. Chem. Soc. Rev..

[2] Ernst M., Rostislav F., Calzolari A., Grieser F. F., Ber S., Gryn’ova G. (2025). Fragment to Framework: Bottom-Up Engineering of Band
Gaps in Covalent Organic Frameworks. ChemRxiv
Preprint.

[3] Prakash K., Deka R., Mobin S. M. (2024). A review
on covalent organic frameworks:
exploration of their growing potential as porous materials in photocatalytic
applications. Inorganic Chemistry Frontiers.

[4] Boland C. S., Sun Y., Papageorgiou D. G. (2024). Bandgap
Engineering of 2D Materials
toward High-Performing Straintronics. Nano Lett..

[5] Oh Y., Song S., Bae J. (2024). A review of
bandgap engineering and
prediction in 2D material heterostructures: a DFT perspective. International Journal of Molecular Sciences.

[6] Liu Q., Li Q., Li Y., Su T., Hou B., Zhao Y., Xu Y. (2025). Two-dimensional Covalent
Organic Frameworks in Organic Electronics. Angew.
Chem., Int. Ed..

[7] Chaves A., Azadani J. G., Alsalman H., Da Costa D., Frisenda R., Chaves A., Song S. H., Kim Y. D., He D., Zhou J. (2020). Bandgap engineering of two-dimensional semiconductor
materials. npj 2D Materials and Applications.

[8] Rahman M. A., Thakur S., Hopkins P. E., Giri A. (2023). Engineering the Electronic
and Thermal Properties of Two-Dimensional Covalent Organic Frameworks. J. Phys. Chem. C.

[9] Singh N., Yadav D., Mulay S. V., Kim J. Y., Park N. J., Baeg J. O. (2021). Band Gap Engineering in Solvochromic
2D Covalent Organic
Framework Photocatalysts for Visible Light-Driven Enhanced Solar Fuel
Production from Carbon Dioxide. ACS Appl. Mater.
Interfaces.

[10] Benedetto G., Mirica K. A. (2024). Conductive Framework Materials for Chemiresistive Detection
and Differentiation of Toxic Gases. Acc. Chem.
Res..

[11] Jain C., Kushwaha R., Rase D., Shekhar P., Shelke A., Sonwani D., Ajithkumar T. G., Vinod C. P., Vaidhyanathan R. (2024). Tailoring
COFs: Transforming Nonconducting 2D Layered COF into a Conducting
Quasi-3D Architecture via Interlayer Knitting with Polypyrrole. J. Am. Chem. Soc..

[12] Wang M., Wang M., Lin H. H., Ballabio M., Zhong H., Bonn M., Zhou S., Heine T., Cánovas E., Dong R. (2020). High-Mobility Semiconducting Two-Dimensional Conjugated
Covalent Organic Frameworks with p-Type Doping. J. Am. Chem. Soc..

[13] Krishnaveni V., DMello M. E., Sahoo P., Thokala N., Bakuru V. R., Vankayala K., Basavaiah K., Kalidindi S. B. (2023). Palladium-Nanoparticle-Decorated
Covalent Organic Framework Nanosheets for Effective Hydrogen Gas Sensors. ACS Applied Nano Materials.

[14] Meng Z., Stolz R. M., Mirica K. A. (2019). Two-Dimensional
Chemiresistive Covalent
Organic Framework with High Intrinsic Conductivity. J. Am. Chem. Soc..

[15] Wang M., Fu S., Petkov P., Fu Y., Zhang Z., Liu Y., Ma J., Chen G., Gali S. M., Gao L. (2023). Exceptionally
high charge mobility in phthalocyanine-based poly (benzimidazobenzophenanthroline)-ladder-type
two-dimensional conjugated polymers. Nat. Mater..

[16] Lu M., Li Q., Liu J., Zhang F.-M., Zhang L., Wang J.-L., Kang Z.-H., Lan Y.-Q. (2019). Installing earth-abundant metal active
centers to covalent organic frameworks for efficient heterogeneous
photocatalytic CO2 reduction. Applied Catalysis
B: Environmental.

[17] Cai S., Sun B., Li X., Yan Y., Navarro A., Garzón-Ruiz A., Mao H., Chatterjee R., Yano J., Zhu C. (2020). Reversible
Interlayer Sliding and Conductivity Changes in Adaptive Tetrathiafulvalene-Based
Covalent Organic Frameworks. ACS Appl. Mater.
Interfaces.

[18] Tong M., Lan Y., Yang Q., Zhong C. (2017). Exploring the structure-property
relationships of covalent organic frameworks for noble gas separations. Chem. Eng. Sci..

[19] Pütz A. M., Terban M. W., Bette S., Haase F., Dinnebier R. E., Lotsch B. V. (2020). Total scattering reveals the hidden
stacking disorder
in a 2D covalent organic framework. Chemical
Science.

[20] Gao F., Ding Z., Meng S. (2013). Three-dimensional
metal-intercalated
covalent organic frameworks for near-ambient energy storage. Sci. Rep..

[21] Pakhira S., Lucht K. P., Mendoza-Cortes J. L. (2017). Iron intercalation
in covalent-organic
frameworks: A promising approach for semiconductors. J. Phys. Chem. C.

[22] Pakhira S., Mendoza-Cortes J. L. (2019). Intercalation of first row transition
metals inside
covalent-organic frameworks (COFs): A strategy to fine tune the electronic
properties of porous crystalline materials. Phys. Chem. Chem. Phys..

[23] Sinha N., Pakhira S. (2021). Tunability of the electronic properties of covalent
organic frameworks. ACS Applied Electronic Materials.

[24] Sinha N., Joshi H., Pakhira S. (2022). Lithium intercalation
in covalent
organic frameworks: a porous electrode material for lithium-ion batteries. ACS Applied Electronic Materials.

[25] Maldonado-Lopez D., Cortes J. L. M. (2023). Exquisite control
of electronic and spintronic properties
on highly porous covalent organic frameworks (COFs): Transition metal
intercalation in bilayers. Phys. Scr..

[26] Shi J.-L., Feng K., Hao H., Ku C., Sit P. H.-L., Teoh W. Y., Lang X. (2022). 2D sp2 Carbon-Conjugated
Covalent
Organic Framework with Pyrene-Tethered TEMPO Intercalation for Photocatalytic
Aerobic Oxidation of Sulfides into Sulfoxides. Solar RRL.

[27] Shen R., Li X., Qin C., Zhang P., Li X. (2023). Efficient Photocatalytic
Hydrogen Evolution by Modulating Excitonic Effects in Ni-Intercalated
Covalent Organic Frameworks. Adv. Energy Mater..

[28] Wang H., Zou W., Liu C., Sun Y., Xu Y., Sun W., Wang Y. (2023). *β*-Ketoenamine-Linked covalent organic framework
with Co intercalation: improved lithium-storage properties and mechanism
for high-performance lithium-organic batteries. Batteries & Supercaps.

[29] Poręba T., Ernst M., Zimmer D., Macchi P., Casati N. (2019). Pressure-Induced
Polymerization and Electrical Conductivity of a Polyiodide. Angew. Chem., Int. Ed..

[30] Sun J., Iakunkov A., Baburin I. A., Joseph B., Palermo V., Talyzin A. V. (2020). Covalent Organic Framework (COF-1) under High Pressure. Angew. Chem..

[31] Fang J., Fu Z., Chen X., Liu Y., Chen F., Wang Y., Li H., Yusran Y., Wang K., Valtchev V. (2023). Piezochromism
in Dynamic Three-Dimensional Covalent Organic Frameworks. Angew. Chem., Int. Ed..

[32] Erkartal M. (2023). Unveiling
the multifaceted properties of a 3d covalent-organic framework: Pressure-induced
phase transition, negative linear compressibility and auxeticity. Comput. Mater. Sci..

[33] Kanon K., Sharif S. S., Irfan A., Sharif A. (2024). Inorganic film materials
for flexible electronics: A brief overview, properties, and applications. Engineering Reports.

[34] Tantardini C., Kvashnin A. G., Gatti C., Yakobson B. I., Gonze X. (2021). Computational
modeling of 2D materials under high pressure and their chemical bonding:
Silicene as possible field-effect transistor. ACS Nano.

[35] Lukose B., Kuc A., Heine T. (2011). The structure of layered covalent-organic frameworks. Chem.—Eur. J..

[36] Sahnoun R., Mijoule C. (2001). Density functional study of metal- arene compounds:
mono (benzene) chromium, bis (benzene) chromium and their cations. J. Phys. Chem. A.

[37] Furness J. W., Kaplan A. D., Ning J., Perdew J. P., Sun J. (2020). Accurate and
numerically efficient r2SCAN meta-generalized gradient approximation. J. Phys. Chem. Lett..

[38] VandeVondele J., Krack M., Mohamed F., Parrinello M., Chassaing T., Hutter J. (2005). Quickstep: Fast and accurate density
functional calculations using a mixed Gaussian and plane waves approach. Comput. Phys. Commun..

[39] VandeVondele J., Hutter J. (2007). Gaussian basis sets for accurate
calculations on molecular
systems in gas and condensed phases. J. Chem.
Phys..

[40] Krack M. (2005). Pseudopotentials
for H to Kr optimized for gradient-corrected exchange-correlation
functionals. Theor. Chem. Acc..

[41] Kühne T. D., Iannuzzi M., Del Ben M., Rybkin V. V., Seewald P., Stein F., Laino T., Khaliullin R. Z., Schütt O., Schiffmann F. (2020). CP2K: An electronic
structure and molecular dynamics software package-Quickstep: Efficient
and accurate electronic structure calculations. J. Chem. Phys..

[42] Du G., Zhao L., Li S., Huang J., Fang S., Han W., Li J., Du Y., Ming J., Zhang T. (2025). Interlayer engineering
of lattice dynamics and elastic constants
of 2D layered nanomaterials under pressure. Nat. Commun..

[43] Pogna E. A., Bosak A., Chumakova A., Milman V., Winkler B., Viti L., Vitiello M. S. (2022). Lattice dynamics and elastic properties
of black phosphorus. Phys. Rev. B.

[44] Hanfland M., Beister H., Syassen K. (1989). Graphite under
pressure: Equation
of state and first-order Raman modes. Phys.
Rev. B.

[45] Rêgo C. R., Oliveira L. N., Tereshchuk P., Da Silva J. L. (2015). Comparative study
of van der Waals corrections to the bulk properties of graphite. J. Phys.: Condens. Matter.

[46] Feng G., Qin Y., Ran C., Ji L., Dong L., Li W. (2018). Structural
evolution and photoluminescence properties of a 2D hybrid perovskite
under pressure. APL Materials.

[47] Gupta Y., Rathore S., Singh A., Kumar A. (2022). Tailoring the mechanical
response of Ruddlesden Popper lead halide perovskites. J. Alloys Compd..

[48] Ling S., Slater B. (2015). Unusually large band gap changes in breathing metal–organic
framework materials. J. Phys. Chem. C.

[49] Li Q., Yin L., Chen Z., Deng K., Luo S., Zou B., Wang Z., Tang J., Quan Z. (2019). High pressure structural
and optical properties of two-dimensional hybrid halide perovskite
(CH3NH3) 3Bi2Br9. Inorg. Chem..

[50] Zhang L., Wu L., Wang K., Zou B. (2019). Pressure-induced broadband emission
of 2D organic–inorganic hybrid perovskite (C6H5C2H4NH3) 2PbBr4. Advanced Science.

[51] Yuan Y., Liu X.-F., Ma X., Wang X., Li X., Xiao J., Li X., Zhang H.-L., Wang L. (2019). Large band
gap narrowing and prolonged carrier lifetime of (C4H9NH3) 2PbI4 under
high pressure. Advanced Science.

[52] Geng T., Ma Z., Chen Y., Cao Y., Lv P., Li N., Xiao G. (2020). Bandgap engineering
in two-dimensional halide perovskite Cs 3 Sb
2 I 9 nanocrystals under pressure. Nanoscale.

[53] Zhao Z., Zhang H., Yuan H., Wang S., Lin Y., Zeng Q., Xu G., Liu Z., Solanki G., Patel K. (2015). Pressure induced metallization
with absence of structural
transition in layered molybdenum diselenide. Nat. Commun..

[54] Nayak A. P., Yuan Z., Cao B., Liu J., Wu J., Moran S. T., Li T., Akinwande D., Jin C., Lin J.-F. (2015). Pressure-modulated conductivity, carrier density, and
mobility of multilayered tungsten disulfide. ACS Nano.

[55] Yan Z., Li N., Wang L., Yu Z., Li M., Zhang J., Li X., Yang K., Gao G., Wang L. (2020). Pressure-Induced Two-Color
Photoluminescence and Phase Transition of Two-Dimensional Layered
MnCl2. J. Phys. Chem. C.

[56] Yan Z., Yin K., Yu Z., Li X., Li M., Yuan Y., Li X., Yang K., Wang X., Wang L. (2020). Pressure-induced band-gap
closure and metallization in two-dimensional transition metal halide
CdI2. Applied Materials Today.

[57] Harms N. C., Kim H.-S., Clune A. J., Smith K. A., O’Neal K. R., Haglund A. V., Mandrus D. G., Liu Z., Haule K., Vanderbilt D., Musfeldt J. L. (2020). Piezochromism in
the magnetic chalcogenide
MnPS3. npj Quantum Materials.

[58] Liu H., Du Y., Deng Y., Ye P. D. (2015). Semiconducting black
phosphorus:
synthesis, transport properties and electronic applications. Chem. Soc. Rev..

[59] Chen L., Sun J., Liang J., Qian Z., Dai X., Sun X., Lv X. (2022). Electronic, optical properties and band-gap tunability of monolayer
antimony under pressure: A first-principle study. Vacuum.

[60] Ke F., Chen Y., Yin K., Yan J., Zhang H., Liu Z., Tse J. S., Wu J., Mao H.-k., Chen B. (2019). Large bandgap
of pressurized trilayer graphene. Proc. Natl.
Acad. Sci. U. S. A..

[61] Lu C., Clayville B., Choi J. Y., Park J. (2023). 2D metal-organic frameworks
as an emerging platform with tunable electronic structures. Chem..

[62] Rajapakse M., Karki B., Abu U. O., Pishgar S., Musa M. R. K., Riyadh S. S., Yu M., Sumanasekera G., Jasinski J. B. (2021). Intercalation as a versatile tool for fabrication,
property tuning, and phase transitions in 2D materials. npj 2D Materials and Applications.

[63] Wan J., Lacey S. D., Dai J., Bao W., Fuhrer M. S., Hu L. (2016). Tuning two-dimensional nanomaterials by intercalation: materials,
properties and applications. Chem. Soc. Rev..

[64] Cao Q., Grote F., Huβmann M., Eigler S. (2021). Emerging field of few-layered
intercalated 2D materials. Nanoscale Advances.

[65] Lukose B., Kuc A., Frenzel J., Heine T. (2010). On the reticular construction concept
of covalent organic frameworks. Beilstein journal
of nanotechnology.

[66] Kuc A., Springer M. A., Batra K., Juarez-Mosqueda R., Wöll C., Heine T. (2020). Proximity Effect in Crystalline Framework
Materials: Stacking-Induced Functionality in MOFs and COFs. Adv. Funct. Mater..

