# Supplementary material for: Extensive Band Gap Tunability in Covalent Organic Frameworks via Metal Intercalation and High Pressure
Source: J Phys Chem Lett. 2025 Jul 15;16(29):7398–405. doi: 10.1021/acs.jpclett.5c01216 (PMC12302217; doi:10.1021/acs.jpclett.5c01216)
Supplement: Supplementary file 1 [file jz5c01216_si_001.pdf]

Extensive Band Gap Tunability in Covalent Organic Frameworks via Metal Intercalation and High Pressure

Michelle Ernst,[*,†,‡] Jürg Hutter,[†] and Stefano Battaglia[†]

†*Department of Chemistry, University of Zurich, 8057 Zürich, Switzerland.*

‡*Current address: Institute of Geological Sciences, University of Bern, Baltzerstrasse 1+3, 3012 Bern, Switzerland.*

E-mail: michelle.ernst@unibe.ch

1 Computational details and parameter selection

1.1 Cell optimization COF-1 at 0 GPa

Table S1 provides an overview of calculations performed for pristine COF-1 at 0 GPa using different computational settings and different functionals (PBE,[1] with and without the D3 correction[2]), highlighting their impact on structural parameters, total energy, and computational cost. Unless otherwise noted, the DZVP-MOLOPT-SR-GTH basis set[3] was used throughout.

Table S1: Summary of cell optimization results for COF-1 at 0 GPa using different computational settings. In bold what was used to obtain the results reported in the main part.

k-points	method	a,b	c	Cell optimization steps	energy [Hartree]	CPU time
1x1x1	r^{2}SCAN	15.072	7.350	6	-445.765	6621.892
1x1x3	r^{2}SCAN	15.076	6.849	4	-445.784	4584.411
1x1x6	r^{2}SCAN	15.075	6.846	5	-445.784	5365.925
1x1x7	r^{2}SCAN	15.075	6.846	5	-445.784	5272.849
1x1x13	r^{2}SCAN	15.075	6.846	5	-445.784	8053.982
**3x3x7**	**r^{2}SCAN**	15.072	6.851	6	-445.785	11552.61
5x5x7	r^{2}SCAN	15.072	6.851	6	-445.785	19760.76
3x3x7	PBE	15.151	7.719	25	-446.321	39678.93
3x3x7	PBE-D3	15.108	6.718	7	-446.524	14323.80
3x3x7	r^{2}SCAN/TZVP	15.066	6.748	4	-445.737	22170.17

As shown by Kothakonda et al.,[4] the inclusion of dispersion corrections has only a minor effect on results obtained with r^{2}SCAN. In our case, the r^{2}SCAN results lead to a unit cell in good agreement with those obtained using PBE-D3.

1.2 Exchange-correlation functionals comparison

Calculations with a hybrid functional such as HSE06[5] in combination with the DZVP-MOLOPT-SR-GTH basis set used in this work would prevent us to sample the Brillouin zone,

as it is computationally too expensive. Therefore, we chose to use the meta-GGA r^{2}SCAN exchange-correlation functional,[6] which provides good accuracy for electronic, structural and thermochemical properties of solid-state materials and transition metal compounds at an affordable computational cost.[4,7–9] Nevertheless, to provide further evidence that r^{2}SCAN provides results in line with more common functionals such as PBE[1] and HSE06,[5] we have computed the gap at the Γ point at 0 GPa, sampling only the Γ point (for HSE06 to be computationally feasible). First, we compare in Table S2 the gap at the Γ point obtained with the electron densities calculated with the 3x3x7 grid and a 1x1x1 grid, respectively, using the r^{2}SCAN functional. As we can see from the last column of Table S2, computing

Table S2: Band gap and gap at the Γ point obtained with the electron densities calculated with the 3x3x7 and 1x1x1 k-point grids, respectively, using the r^{2}SCAN functional. All values are in eV.

k-points<br>System	3x3x7<br>Band gap	3x3x7<br>Gap at Γ	1x1x1<br>Gap at Γ	$\Delta(\Gamma - 3x3x7)$
COF-1	3.33	3.35	3.34	−0.01
COF-1-Ca(0)	0.45	0.95	0.94	−0.01
COF-1-Ca(II)	0.98	1.00	1.03	0.03
COF-1-Cr(0)	0.78	0.79	0.81	0.02
COF-1-Fe(II)	0.08	0.38	0.22	−0.16
COF-1-Fe(III)	0.00	0.02	0.01	−0.01
COF-1-Zn(0)	0.50	1.36	1.31	−0.05
COF-1-Zn(II)	0.90	0.94	0.91	−0.03

the electron density with a 1x1x1 grid provides a reasonable estimate for the gap at Γ compared to the Γ point gap obtained with the 3x3x7 k-point grid. In Table S3 we present the gaps at Γ obtained with HSE06 and PBE, and compare them to the r^{2}SCAN values; in all these cases we calculated the gap with a 1x1x1 grid for a fair comparison. As can be seen, HSE06 systematically results in larger gaps, as expected from the inclusion of exact exchange, whereas PBE systematically results in lower gaps, as expected from the delocalization error typical of GGA functionals. In most cases, HSE06 increases the gap by 0.5 eV to 0.8 eV, with two exceptions, COF-1-Cr(0) and COF-1-Fe(II), where the change is larger than 1 eV. All gaps obtained with PBE are within 0.4 eV from r^{2}SCAN besides COF-1-Ca(II), which

Table S3: Gaps at the Γ point obtained with different functionals. All values are in eV.

System	r^2SCAN	HSE06	PBE	Δ(HSE06-r^2SCAN)	Δ(PBE-r^2SCAN)
COF-1	3.34	4.11	3.05	0.77	−0.29
COF-1-Ca(0)	0.94	1.38	0.88	0.44	−0.06
COF-1-Ca(II)	1.03	1.80	0.05	0.77	−0.98
COF-1-Cr(0)	0.81	2.31	0.40	1.50	−0.41
COF-1-Fe(II)	0.22	1.34	0.19	1.12	−0.03
COF-1-Fe(III)	0.01	n.a.	0.01	n.a	0.00
COF-1-Zn(0)	1.31	1.82	1.15	0.51	−0.16
COF-1-Zn(II)	0.91	1.68	0.74	0.77	−0.17

is smaller by 0.98 eV. More interesting than simply looking at the differences in the gaps between functionals, is the effect that metal intercalation has on the gap predicted by the same functional, which we report in Table S4. As we can see, the effect of metal intercalation

Table S4: Change of the gap at the Γ point due to metal intercalation for different functionals. All values are in eV.

System	r^2SCAN	HSE06	PBE	Δ(HSE06-r^2SCAN)	Δ(PBE-r^2SCAN)
COF-1-Ca(0)	2.40	2.73	2.16	0.33	−0.24
COF-1-Ca(II)	2.31	2.31	3.00	0.00	0.69
COF-1-Cr(0)	2.53	1.80	2.65	−0.73	0.12
COF-1-Fe(II)	3.12	2.77	2.85	−0.35	−0.27
COF-1-Fe(III)	3.33	n.a.	3.04	n.a	−0.29
COF-1-Zn(0)	2.03	2.29	1.90	0.26	−0.13
COF-1-Zn(II)	2.43	2.43	2.30	0.00	−0.13

is similar regardless of the functional used: the range of gap reduction (without accounting for Fe(III) which makes the system metallic) for r^2SCAN is between 2.03 eV and 3.12 eV, for HSE06 this range is between 1.80 eV and 2.77 eV, and for PBE is between 1.90 eV and 3.0 eV. This comparison provides evidence that the trends obtained in this work with r^2SCAN are qualitatively correct.

1.3 Charge and spin states

For COF-1-Cr(0), COF-1-Fe(II), and COF-1-Fe(III) the spin state is ambiguous. Therefore, structural optimizations at 0 GPa were performed with all possible spin multiplicities. The corresponding total energies are given in Table S5.

Table S5: Energies for possible multiplicities.

COF-1-M	Multiplicity	Energy [Hartree]
COF-1-Cr(0)	1	-532.550
COF-1-Cr(0)	3	-532.540
COF-1-Cr(0)	5	-532.536
COF-1-Cr(0)	7	-532.519
COF-1-Fe(II)	1	-569.190
COF-1-Fe(II)	3	-569.174
COF-1-Fe(II)	5	-569.170
COF-1-Fe(III)	2	-569.159
COF-1-Fe(III)	4	-569.124

Since in all cases the lowest multiplicity leads to the lowest energy, only that spin state was used for all subsequent calculations. Table S6 provides an overview of the formal oxidation states and lowest-energy spin states.

Table S6: Intercalated metals, their formal oxidation states, and corresponding most stable spin states.

Compound	Formal oxidation state	Spin
COF-1-Ca	0	Singlet
COF-1-Ca	II	Singlet
COF-1-Cr	0	Singlet
COF-1-Fe	II	Singlet
COF-1-Fe	III	Doublet
COF-1-Zn	0	Singlet
COF-1-Zn	II	Singlet

Charged systems were calculated using a constant neutralizing background charge for the electrostatic energy.

2 Geometric features of intercalated COFs

2.1 Unit cell parameters

Table S7 shows the unit cell parameters of the COF-1(-M). For the pristine COF-1 the calculations were done up to 10 GPa for the intercalated systems up to 5 GPa.

Table S7: Optimized unit cell dimensions and volumes of COF-1-M at different pressures

	Pressure [GPa]	Unit cell dimensions [Å]	Volume [Å^3]
COF-1	0	a=15.07, b=15.07, c=6.85	1347.82
COF-1	2.5	a=14.88, b=14.88, c=6.00	1150.83
COF-1	5	a=14.73, b=14.73, c=5.68	1067.37
COF-1	7.5	a=14.59, b=14.59, c=5.47	1009.72
COF-1	10.0	a=14.47, b=14.47, c=5.32	964.20
COF-1-Ca(0)	0	a=15.08, b=15.08, c=7.67	1511.95
COF-1-Ca(0)	2.5	a=14.86, b=14.86, c=6.79	1298.51
COF-1-Ca(0)	5	a=14.69, b=14.69, c=6.38	1191.84
COF-1-Ca(II)	0	a=14.93, b=14.93, c=6.22	1201.22
COF-1-Ca(II)	2.5	a=14.79, b=14.79, c=5.57	1055.55
COF-1-Ca(II)	5	a=14.02, b=14.02, c=5.31	904.00
COF-1-Cr(0)	0	a=15.06, b=15.06, c=6.91	1357.62
COF-1-Cr(0)	2.5	a=14.87, b=14.87, c=6.19	1184.81
COF-1-Cr(0)	5	a=14.71, b=14.71, c=5.88	1102.10
COF-1-Fe(II)	0	a=14.95, b=14.95, c=6.34	1233.09
COF-1-Fe(II)	2.5	a=14.76, b=14.76, c=5.91	1114.85
COF-1-Fe(II)	5	a=14.60, b=14.60, c=5.67	1045.62
COF-1-Fe(III)	0	a=14.92, b=14.92, c=6.10	1176.08
COF-1-Fe(III)	2.5	a=14.73, b=14.73, c=5.78	1085.85
COF-1-Fe(III)	5	a=14.56, b=14.56, c=5.57	1022.92
COF-1-Zn(0)	0	a=15.07, b=15.07, c=7.20	1416.13
COF-1-Zn(0)	2.5	a=14.89, b=14.89, c=6.33	1214.05
COF-1-Zn(0)	5	a=14.74, b=14.74, c=5.96	1121.06
COF-1-Zn(II)	0	a=14.98, b=14.98, c=6.56	1275.32
COF-1-Zn(II)	2.5	a=14.78, b=14.78, c=6.10	1154.83
COF-1-Zn(II)	5	a=14.62, b=14.62, c=5.85	1081.92

2.2 Surface area and density

Table S8 shows the total surface area, total helium volume, and density of the COF-1-M structures at 0 GPa estimated with the Pore Analyser tool in Mercury 2024.2.0.[10]

Table S8: Internal surface, volume and density.

COF	Total surface area [$Å^2$]	Total helium volume [$Å^3$]	Density [g/cm^3]
COF-1	109.36	535.44	0.959
COF-1-Ca(0)	139.71	641.75	0.884
COF-1-Ca(II)	86.39	453.86	1.131
COF-1-Cr(0)	123.48	549.82	1.016
COF-1-Fe(II)	103.95	492.00	1.123
COF-1-Fe(III)	98.76	469.91	1.178
COF-1-Zn(0)	115.81	575.35	0.989
COF-1-Zn(II)	100.66	501.81	1.099

2.3 Comparison of cell parameters to experiment

We compared our results of the pristine COF-1 cell parameters to the experimental data reported by Sun et al.,[11] estimating their values from published figures. Based on this, the agreement is excellent (see Figure S1).

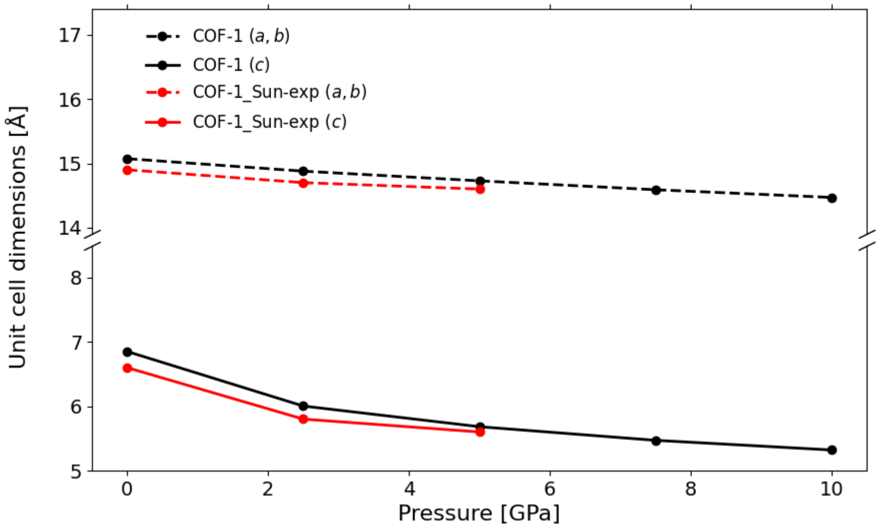

Figure S1: Comparison of cell parameters to experimental results of Sun et al.[11]

2.4 Binding energies and adsorption sites

To calculate the binding energy of the COF-1-M complex (CM), we consider the association of two fragments: the COF-1 framework (C) and the metal atom (M). The binding energy is the sum of the (destabilizing) deformation energy and the (stabilizing) interaction energy.

$$E^{\text{bind}} = E^{\text{def}} + E^{\text{int}} \tag{1}$$

The deformation energy is the sum of the deformation energies of both fragments:

$$E^{\text{def}} = E^{\text{def}}_{\text{C}} + E^{\text{def}}_{\text{M}} = (E_{\text{C}\in\text{CM}} - E_{\text{C}}) + (E_{\text{M}\in\text{CM}} - E_{\text{M}}) \tag{2}$$

The counterpoise-corrected interaction energy is the energy of the optimized complex in its full basis minus the energy of the fragments in the geometry of the complex and also with the basis set of the full complex:

$$E^{\text{int}} = E^{\text{CM basis}}_{\text{CM}} - E^{\text{CM basis}}_{\text{C}\in\text{CM}} - E^{\text{CM basis}}_{\text{M}\in\text{CM}} \tag{3}$$

Inserting this into equation 1 gives:

$$E^{\text{bind}} = (E_{\text{C}\in\text{CM}} - E_{\text{C}}) + (E_{\text{M}\in\text{CM}} - E_{\text{M}}) + \left(E^{\text{CM basis}}_{\text{CM}} - E^{\text{CM basis}}_{\text{C}\in\text{CM}} - E^{\text{CM basis}}_{\text{M}\in\text{CM}}\right) \tag{4}$$

Since in our case the metal atom is just a single atom, we set:

$$E_{\text{M}\in\text{CM}} - E_{\text{M}} = 0 \tag{5}$$

Furthermore for $E^{\text{CM basis}}_{\text{M}\in\text{CM}}$ we chose M to be in a large cubic box of side-length 30 Å with

ghost atoms of the COF. Then the binding energy simplifies to:

$$E^{\text{bind}} = (E_{\text{C}\in\text{CM}} - E_{\text{C}}) + \left(E_{\text{CM}}^{\text{CM basis}} - E_{\text{C}\in\text{CM}}^{\text{CM basis}} - E_{\text{M}\in\text{cubicBox}}^{\text{CM basis}}\right) \tag{6}$$

The binding energies for the charged ions Ca(II) and Fe(II) would result in overestimated values without taking into account the energy needed to dissociate them from the originating counterions. Therefore, we did not calculate the binding energies for these two cases as it would be very arbitrary. Instead, we investigated their site preferences by placing the ions at different initial positions and performing full geometry optimizations. Ca(II) was inserted between the benzene rings and between the boroxine rings, while Fe(II) was additionally placed near an oxygen atom due to its known affinity for oxygen coordination. The resulting total energies were then compared to assess the relative stability of each configuration (*cf.* Table S9). Positive values represent less stabilizing energies.

Table S9: Energies at different adsorption sites

	Metal adsorption site	Energy relative to benzene adsorption site [kJ/mol]
COF-1-Ca(0)	between benzene	0
COF-1-Ca(0)	between boroxine	+5.0
COF-1-Ca(II)	between benzene	0
COF-1-Ca(II)	between boroxine	+17.1
COF-1-Fe(II)	between benzene	0
COF-1-Fe(II)	between boroxine	+364.5
COF-1-Fe(II)	near oxygen	+104.3

2.5 Interlayer distance

Figure S2 displays the interlayer spacing measured in c-direction between two layers containing intercalated metal atoms, and plotted against the corresponding Mulliken charges. The red points indicate the Mulliken charge on the metal atoms, while the blue points correspond to the summed charge of all the layer atoms. The interlayer distance between two adjacent layers without metal intercalation is given by the difference between the unit cell *c*-axis and this value.

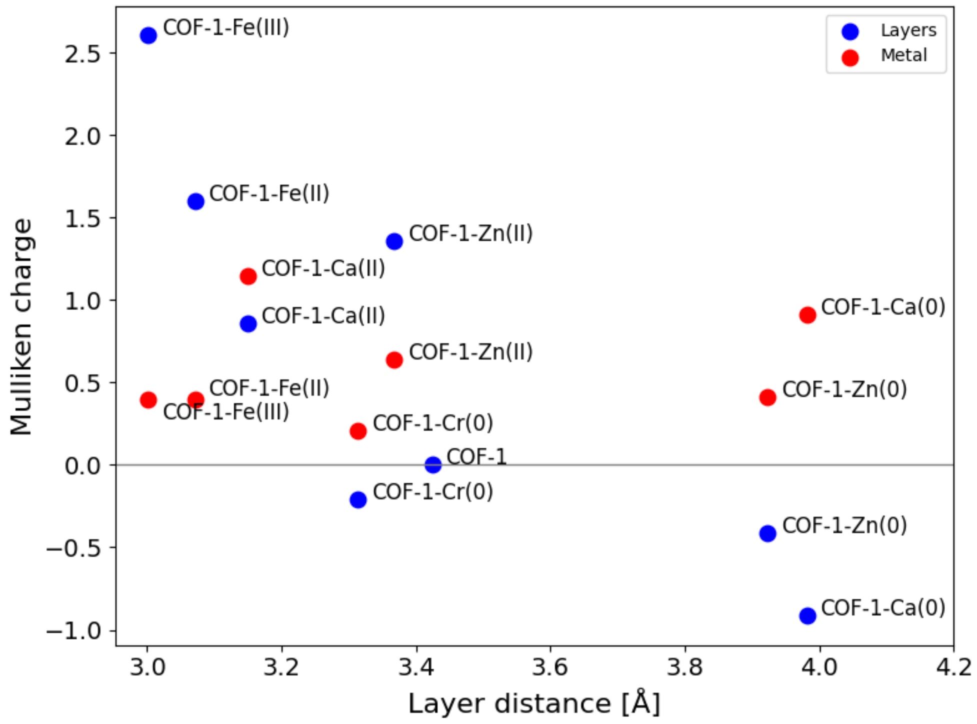

Figure S2: Mulliken charges on the layers above and below the metal center (blue) and on the metal atom itself (red) as a function of the interlayer distance.

2.6 Structural rearrangement COF-1-Ca(II)

At ambient pressure (0 GPa), COF-1-Ca(II) exhibits a structure characterized by laterally shifted 2D layers, indicative of a relatively flexible interlayer interaction. Upon compression to 5 GPa, the framework undergoes a marked structural response: the planar sheets bend out of plane, leading to a pronounced distortion of the originally stacked geometry. This pressure-induced deformation is illustrated in Figure S3.

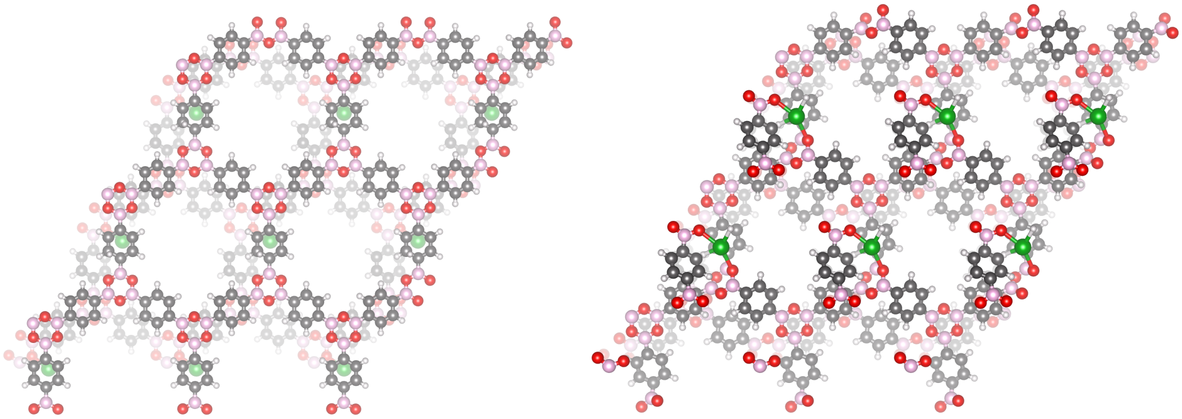

Figure S3: COF-1-Ca(II) at 0 GPa (left) and 5 GPa (right).

3 Electronic structure

Table S10 summarizes the band gaps of all structures.

Table S10: Band gaps of COF-1-M at different pressures

	Pressure [GPa]	Band gap [eV]
COF-1	0	3.33
COF-1	2.5	3.05
COF-1	5	2.86
COF-1	7.5	2.64
COF-1	10	2.31
COF-1-Ca(0)	0	0.45
COF-1-Ca(0)	2.5	0.46
COF-1-Ca(0)	5	0.58
COF-1-Ca(II)	0	0.98
COF-1-Ca(II)	2.5	0.51
COF-1-Ca(II)	5	1.17
COF-1-Cr(0)	0	0.78
COF-1-Cr(0)	2.5	0.65
COF-1-Cr(0)	5	0.52
COF-1-Fe(II)	0	0.08
COF-1-Fe(II)	2.5	0.06
COF-1-Fe(II)	5	0.05
COF-1-Fe(III)	0	0.00
COF-1-Fe(III)	2.5	0.00
COF-1-Fe(III)	5	0.00
COF-1-Zn(0)	0	0.50
COF-1-Zn(0)	2.5	0.70
COF-1-Zn(0)	5	0.63
COF-1-Zn(II)	0	0.90
COF-1-Zn(II)	2.5	0.65
COF-1-Zn(II)	5	0.48

All band gaps are indirect, but some bands are very flat and the direct band gaps are not much smaller than the indirect ones.

Figure S4 depicts the band gaps as ranges.

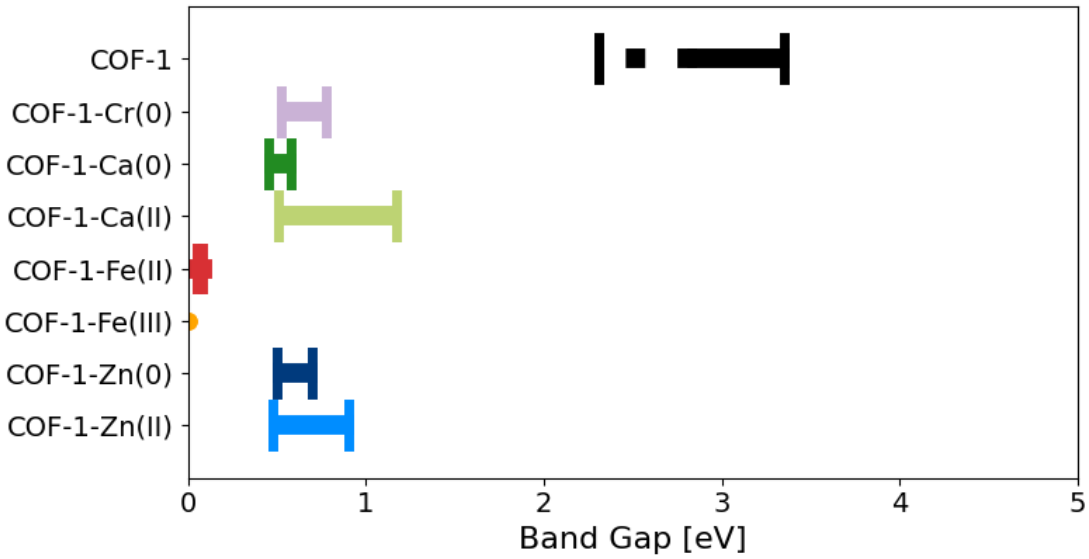

Figure S4: Band gap ranges from 0 GPa to 5 GPa with a continuous line and from 5 GPa to 10 GPa with a dashed line.

Figure S5 shows the band structures calculated for and in-between the special k-points of a hexagonal crystal, as well as the DOS. All scripts used to plot the band structure, DOS and pDOS are available at github.com/stefabat/cp2k-scripts.

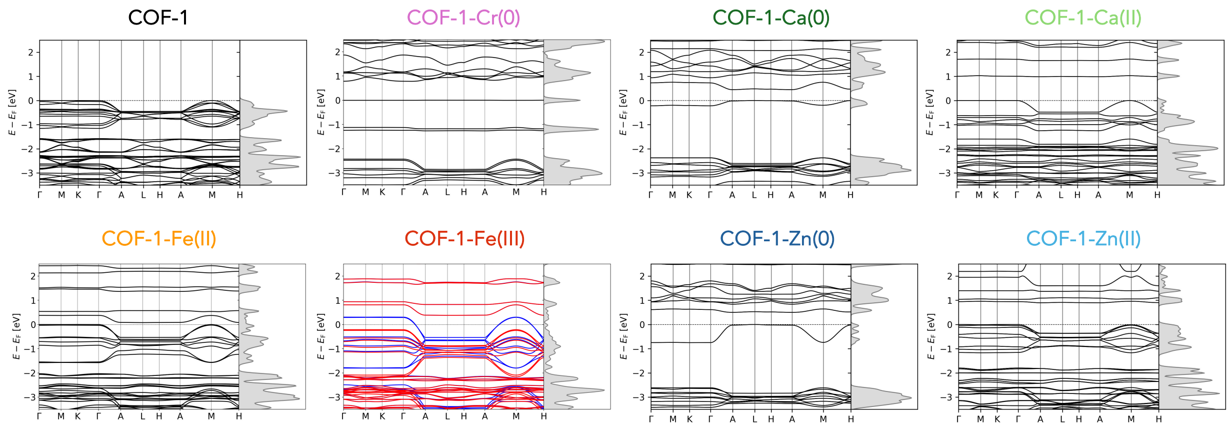

Figure S5: Band structures at 0 GPa. For Fe(III) α-spin is shown in blue, and β-spin in red.

Fe(III) is the only spin-polarized system. At the Γ-point, the two uppermost red bands shown in the plot lie above the Fermi energy due to the use of smearing (300 K). Thus, their

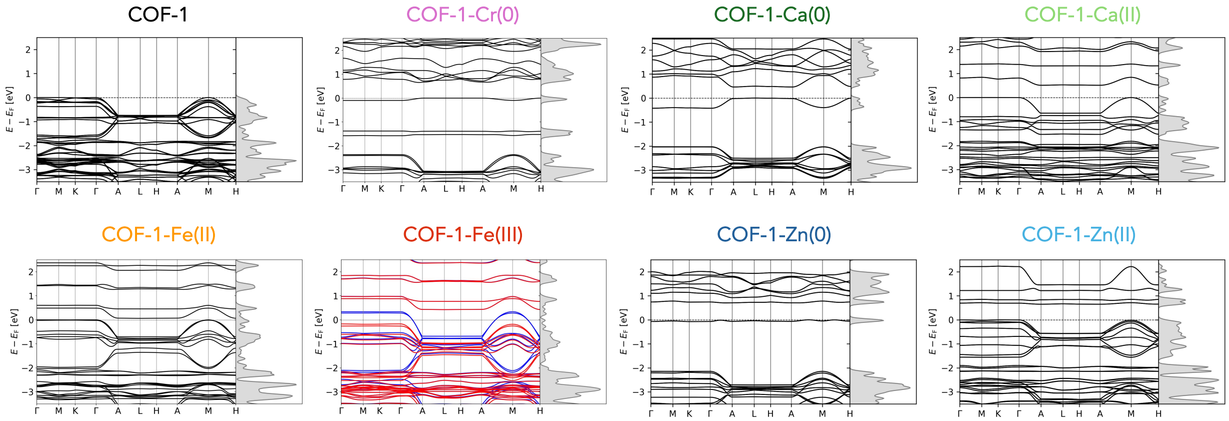

Figure S6: Band structures at 2.5 GPa. For Fe(III) α-spin is shown in blue, and β-spin in red.

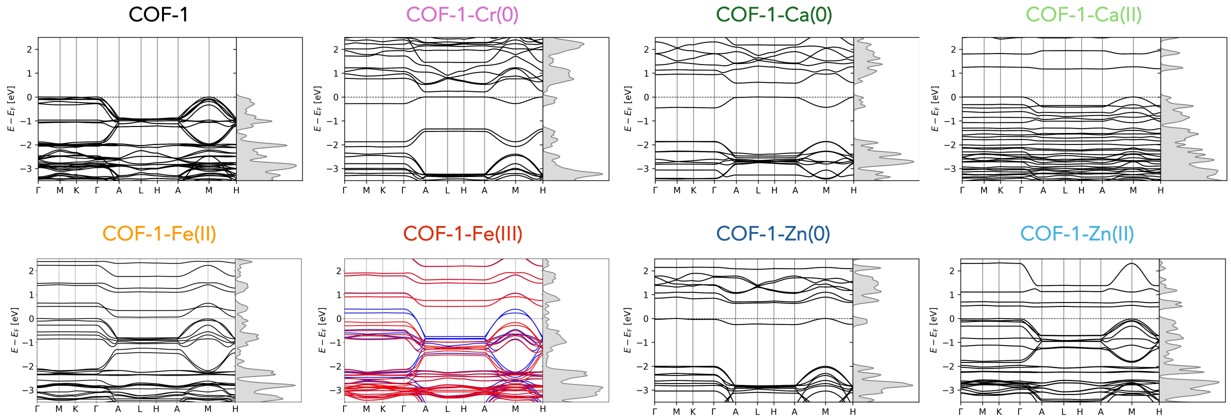

Figure S7: Band structures at 5 GPa. For Fe(III) α-spin is shown in blue, and β-spin in red.

occupation is nearly zero. As a result, the HOCO (*cf.* Figure 3 in main), which in CP2K can only be determined from a Γ-point calculation, corresponds to an unoccupied orbital at some k-points.

4 Triazine-based COF-IITI-0

To complement our analysis, we also examined COF-IITI-0, a structurally distinct framework incorporating the widely used triazine-based linker. This material consists of triboronate ester nodes connected to 2,4,6-triphenyl-1,3,5-triazine units and phenylene rings (*cf.* Figure S8, left) and was computationally proposed by Sinha and Pakhira.[12] Table S11 summarizes the structural and electronic properties obtained from cell optimizations (performed at the same level of theory as for COF-1 with a bilayer unit cell) and band structure calculations at three different pressures.

Table S11: Structural and electronic data for COF-IITI-0

Pressure [GPa]	a,b [Å]	c [Å]	Volume [Å^3]	Band gap [eV]
0	22.29	7.47	3214.78	1.48
2.5	21.93	6.41	2671.32	0.40
4	21.75	6.19	2535.52	0.05

Upon compression, the layers remain AA stacked. With increasing pressure, the band dispersion increases significantly (*cf.* Figure S8, right), indicating enhanced orbital overlap between the layers. This is accompanied by a pronounced decrease in the band gap, leading to near-metallic behavior already at 4 GPa. Compared to COF-1, the band gap decreases more rapidly, likely as a result of the AA stacking geometry (COF-1 has a slight layer offset) rather than differences in chemical composition. For a discussion on how stacking arrangements affect the electronic structure at ambient pressure, we refer to the work of Lukose *et al.*[13]

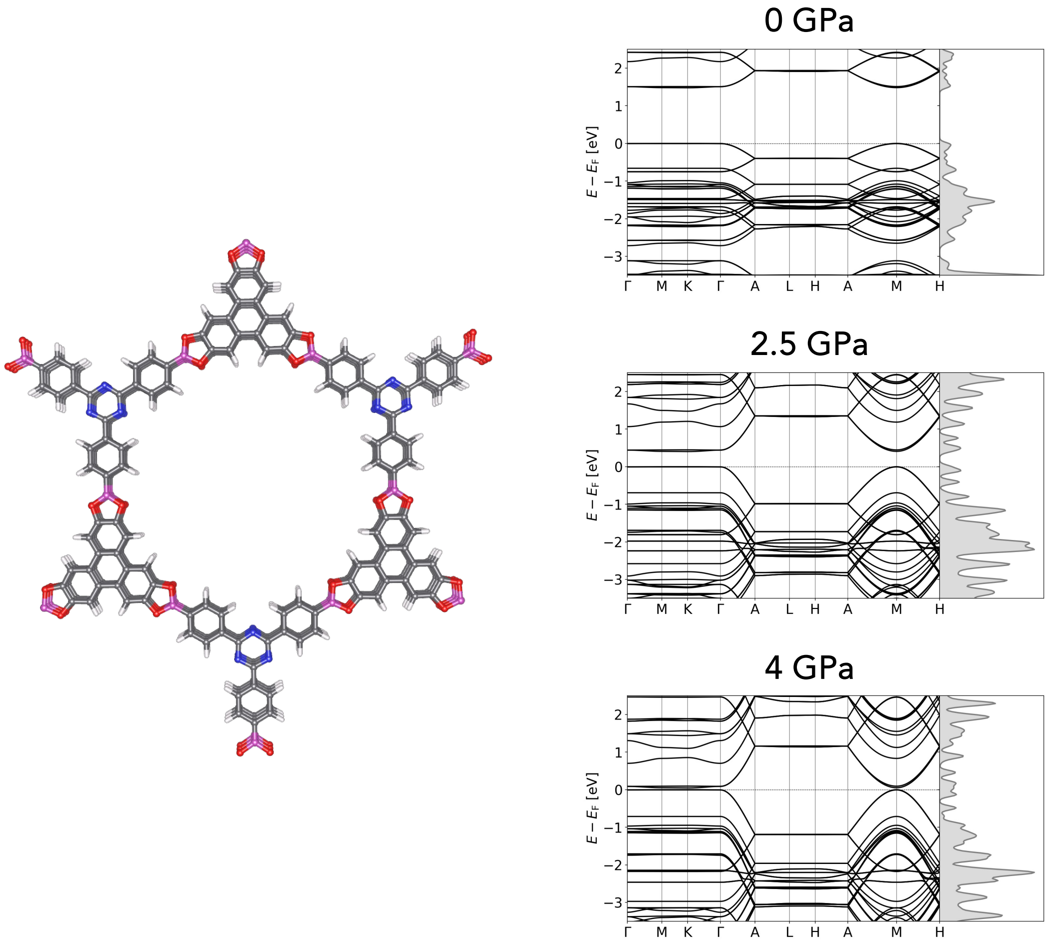

Figure S8: Left: structural model of COF-IITI-0. Right: band structures at 0, 2.5, and 4 GPa, showing progressive band dispersion and band gap narrowing under pressure.

5 Pressure dependence of the band gap in other layered two-dimensional materials

Layered perovskites show a pronounced band gap modulation under pressure. Li *et al.* studied the layered 2D perovskite $MA_3Bi_2Br_9$ (MA = CH_3NH_3) under compression up to 10.4 GPa and observed a band gap reduction from 2.65 eV at ambient pressure to 2.48 eV at 4.6 GPa, prior to a structural phase transition. After a brief increase associated with the transition, the band gap continued to decrease at higher pressures.[14] Similarly, Zhang *et al.* investigated $(C_6H_5C_2H_4NH_3)_2PbBr_4$ and reported a continuous band gap narrowing from 2.96 eV at ambient pressure to 2.46 eV at 12 GPa, where pressure-induced distortions became significant.[15] Yuan *et al.* explored the compression of $(C_4H_9NH_3)_2PbI_4$, observing a steady band gap reduction from 2.28 eV at ambient pressure to 1.25 eV at 27.5 GPa, where the material approached the Shockley–Queisser limit for optimal photovoltaic performance. At the highest pressures measured (approx. 35 GPa), the band gap reached a minimum of approximately 0.95 eV.[16] For a purely inorganic perovskite, Geng *et al.* reported a continuous narrowing of the band gap in 2D $Cs_3Sb_2I_9$ nanocrystals, from 2.05 eV to 1.36 eV under compression up to 20 GPa, with no phase transitions observed.[17]

A similar pressure response has been reported for transition-metal dichalcogenides, such as molybdenum diselenide ($MoSe_2$).[18] Under compression, $MoSe_2$ exhibits a gradual narrowing of the band gap, ultimately leading to metallization. The band gap was first quantitatively extracted at 20.2 GPa, as the optical transmittance spectra below this pressure showed minimal variation, making precise determination unreliable. Complete closure of the band gap and the onset of metallic behavior were observed at approximately 40 GPa. Tungsten disulfide (WS_2) is another layered transition-metal dichalcogenide that exhibits an indirect band gap of approximately 1.3 eV in its multilayered form.[19] Under compression, WS_2 undergoes an isostructural semiconductor-to-metal transition, with metallization occurring around 22 GPa.

Transition-metal dihalides also exhibit significant band gap modulation under compression. For $MnCl_2$, Yan *et al.* reported three structural phase transitions at 4.0, 21.7, and 47.2 GPa. UV–vis absorption spectroscopy revealed a continuous redshift of the absorption edge, with the band gap decreasing from 5.86 eV at ambient pressure to 2.3 eV at 67.4 GPa.[20] In a related study on CdI_2, the band gap narrowed gradually from 3.17 eV at 0.6 GPa to 0.2 eV at 44.6 GPa, with a faster decrease observed beyond 34.5 GPa.[21]

A similar trend is found in transition-metal phosphorus trisulfides. Harms *et al.* reported that the direct band gap of $MnPS_3$ decreases from 2.64 eV at ambient conditions to 2.15 eV at 11.5 GPa, based on optical absorption data fitted using Tauc plots.[22]

Layered black phosphorus as studied by Liu *et al.* also exhibited a pressure sensitivity. In its few-layer form, it features a direct band gap of around 0.3 eV, which increases to over 1 eV in the monolayer. Upon applying compression, the band gap transitions from direct to indirect near 0.6 GPa and closes entirely around 1.7 GPa.[23]

First-principles calculations of Chen *et al.* show that monolayer antimony exhibits an indirect band gap of 1.27 eV at ambient pressure, which decreases continuously under compression and closes entirely at a pressure of 3 GPa, marking a transition from a semiconductor to a metallic state.[24]

References

(1) Perdew, J. P.; Burke, K.; Ernzerhof, M. Generalized Gradient Approximation Made Simple. *Physical Review Letters* **1996**, *77*, 3865–3868.

(2) Grimme, S.; Antony, J.; Ehrlich, S.; Krieg, H. A Consistent and Accurate *Ab Initio* Parametrization of Density Functional Dispersion Correction (DFT-D) for the 94 Elements H-Pu. *The Journal of Chemical Physics* **2010**, *132*, 154104.

(3) VandeVondele, J.; Hutter, J. Gaussian basis sets for accurate calculations on molecular

systems in gas and condensed phases. *The Journal of chemical physics* **2007**, *127*, 114105.

(4) Kothakonda, M.; Kaplan, A. D.; Isaacs, E. B.; Bartel, C. J.; Furness, J. W.; Ning, J.; Wolverton, C.; Perdew, J. P.; Sun, J. Testing the r2SCAN Density Functional for the Thermodynamic Stability of Solids with and without a van der Waals Correction. *ACS Materials Au* **2022**, *3*, 102–111.

(5) Krukau, A. V.; Vydrov, O. A.; Izmaylov, A. F.; Scuseria, G. E. Influence of the Exchange Screening Parameter on the Performance of Screened Hybrid Functionals. *The Journal of Chemical Physics* **2006**, *125*, 224106.

(6) Furness, J. W.; Kaplan, A. D.; Ning, J.; Perdew, J. P.; Sun, J. Accurate and numerically efficient r2SCAN meta-generalized gradient approximation. *The Journal of Physical Chemistry Letters* **2020**, *11*, 8208–8215.

(7) Isaacs, E. B.; Wolverton, C. Performance of the Strongly Constrained and Appropriately Normed Density Functional for Solid-State Materials. *Physical Review Materials* **2018**, *2*, 063801.

(8) Kingsbury, R.; Gupta, A. S.; Bartel, C. J.; Munro, J. M.; Dwaraknath, S.; Horton, M.; Persson, K. A. Performance Comparison of r2SCAN and SCAN metaGGA Density Functionals for Solid Materials via an Automated, High-Throughput Computational Workflow. *Physical Review Materials* **2022**, *6*, 013801.

(9) Zhang, Y.; Ramasamy, A.; Pokharel, K.; Kothakonda, M.; Xiao, B.; Furness, J. W.; Ning, J.; Zhang, R.; Sun, J. Advances and Challenges of SCAN and R^2 SCAN Density Functionals in Transition-Metal Compounds. *WIREs Computational Molecular Science* **2025**, *15*, e70007.

(10) Macrae, C. F.; Sovago, I.; Cottrell, S. J.; Galek, P. T.; McCabe, P.; Pidcock, E.; Plat-

ings, M.; Shields, G. P.; Stevens, J. S.; Towler, M. et al. Mercury 4.0: From visualization to analysis, design and prediction. *Applied Crystallography* **2020**, *53*, 226–235.

(11) Sun, J.; Iakunkov, A.; Baburin, I. A.; Joseph, B.; Palermo, V.; Talyzin, A. V. Covalent Organic Framework (COF-1) under High Pressure. *Angewandte Chemie* **2020**, *132*, 1103–1108.

(12) Sinha, N.; Pakhira, S. Tunability of the electronic properties of covalent organic frameworks. *ACS Applied Electronic Materials* **2021**, *3*, 720–732.

(13) Lukose, B.; Kuc, A.; Frenzel, J.; Heine, T. On the reticular construction concept of covalent organic frameworks. *Beilstein Journal of Nanotechnology* **2010**, *1*, 60–70.

(14) Li, Q.; Yin, L.; Chen, Z.; Deng, K.; Luo, S.; Zou, B.; Wang, Z.; Tang, J.; Quan, Z. High pressure structural and optical properties of two-dimensional hybrid halide perovskite (CH3NH3) 3Bi2Br9. *Inorganic Chemistry* **2019**, *58*, 1621–1626.

(15) Zhang, L.; Wu, L.; Wang, K.; Zou, B. Pressure-induced broadband emission of 2D organic–inorganic hybrid perovskite (C6H5C2H4NH3) 2PbBr4. *Advanced Science* **2019**, *6*, 1801628.

(16) Yuan, Y.; Liu, X.-F.; Ma, X.; Wang, X.; Li, X.; Xiao, J.; Li, X.; Zhang, H.-L.; Wang, L. Large band gap narrowing and prolonged carrier lifetime of (C4H9NH3) 2PbI4 under high pressure. *Advanced Science* **2019**, *6*, 1900240.

(17) Geng, T.; Ma, Z.; Chen, Y.; Cao, Y.; Lv, P.; Li, N.; Xiao, G. Bandgap engineering in two-dimensional halide perovskite Cs 3 Sb 2 I 9 nanocrystals under pressure. *Nanoscale* **2020**, *12*, 1425–1431.

(18) Zhao, Z.; Zhang, H.; Yuan, H.; Wang, S.; Lin, Y.; Zeng, Q.; Xu, G.; Liu, Z.; Solanki, G.; Patel, K. et al. Pressure induced metallization with absence of structural transition in layered molybdenum diselenide. *Nature communications* **2015**, *6*, 7312.

(19) Nayak, A. P.; Yuan, Z.; Cao, B.; Liu, J.; Wu, J.; Moran, S. T.; Li, T.; Akinwande, D.; Jin, C.; Lin, J.-F. Pressure-modulated conductivity, carrier density, and mobility of multilayered tungsten disulfide. *ACS nano* **2015**, *9*, 9117–9123.

(20) Yan, Z.; Li, N.; Wang, L.; Yu, Z.; Li, M.; Zhang, J.; Li, X.; Yang, K.; Gao, G.; Wang, L. Pressure-Induced Two-Color Photoluminescence and Phase Transition of Two-Dimensional Layered MnCl2. *The Journal of Physical Chemistry C* **2020**, *124*, 23317–23323.

(21) Yan, Z.; Yin, K.; Yu, Z.; Li, X.; Li, M.; Yuan, Y.; Li, X.; Yang, K.; Wang, X.; Wang, L. Pressure-induced band-gap closure and metallization in two-dimensional transition metal halide CdI2. *Applied Materials Today* **2020**, *18*, 100532.

(22) Harms, N. C.; Kim, H.-S.; Clune, A. J.; Smith, K. A.; O'Neal, K. R.; Haglund, A. V.; Mandrus, D. G.; Liu, Z.; Haule, K.; Vanderbilt, D. et al. Piezochromism in the magnetic chalcogenide MnPS3. *npj Quantum Materials* **2020**, *5*, 56.

(23) Liu, H.; Du, Y.; Deng, Y.; Ye, P. D. Semiconducting black phosphorus: synthesis, transport properties and electronic applications. *Chemical Society Reviews* **2015**, *44*, 2732–2743.

(24) Chen, L.; Sun, J.; Liang, J.; Qian, Z.; Dai, X.; Sun, X.; Lv, X. Electronic, optical properties and band-gap tunability of monolayer antimony under pressure: A first-principle study. *Vacuum* **2022**, *206*, 111484.
