# Supplementary material for: Extensive Band Gap Tunability in Covalent Organic Frameworks via Metal Intercalation and High Pressure
Source: J Phys Chem Lett. 2025 Jul 15;16(29):7398–405. doi: 10.1021/acs.jpclett.5c01216 (PMC12302217; doi:10.1021/acs.jpclett.5c01216)
Supplement: Supplementary file 2 [file jz5c01216_si_002.pdf]

jz-2025-01216g.R1

Name: Peer Review Information for "Extensive Band Gap Tunability in Covalent Organic Frameworks via Metal Intercalation and High Pressure"

First Round of Reviewer Comments

Reviewer: 1

Comments to the Author

General Comment:

The present manuscript presents a timely and well-executed computational study on the tuning of covalent organic framework (COF) electronic properties via two distinct mechanisms: high pressure and metal intercalation. The metal intercalation is not new at all and there are several studies which the authors mentioned. Moreover, the authors claimed that the band gap of pristine COF-1 decreases by ~1 eV under compression up to 10 GPa. It is really very very high pressure and it is not easily possible to generate the pressure in experiments and the COF-1 may dissemble/dissociate and break at the 10GPa. After reviewing the manuscript on band gap tuning in metal-intercalated (Ca, Cr, Zn, and Fe) COF-1 materials, I found that this preliminary investigation of electronic properties requires substantial improvement before publication. While the authors examine intercalation and pressure effects on the electronic band gap modulation, the study lacks depth and leaves several critical questions unanswered. Publication should only be considered after the authors address the following comments in a comprehensive manner.

1. This study exclusively focused on COF-1 (with boroxine and benzene building units), limiting its scope. The authors should expand their investigation to include COFs with alternative building units, particularly triazine-based structures. A comparative analysis examining the effects of intercalation and high pressure across these different COFs would reveal which structural motifs yield the most favorable outcomes.

2. The reason of choosing r2SCAN functional DFT method is not mentioned anywhere. In general, HSE06-D method provides better electronic band gap than any other DFT

method. It is also strange that the authors use both Gaussian and plane wave approaches. The reason is not clear at all.

3. The authors wrote that they have computed “Molecular orbitals”. Is it really true? It seems that they have computed 3D band structures then why “Molecular orbitals”?

4. Authors claimed that “all intercalated systems exhibit relatively low bulk moduli compared to other 2D materials”; to claim this point, there should be a proper literature review and references.

5. In Figure 1b, it is reported that the intercalated atom binds to the benzene ring. The author must justify the reason behind choosing the benzene ring for metal intercalation. Site selection could not be decided without checking the stability of the structure. I suggest that authors should calculate the binding energy of metal interaction with all the available active sites on the COF framework.

6. Gao et al. also studied the Ca-intercalated COF-1 (See; Scientific Reports, Volume 3, Article number: 1882 (Year 2013) and https://www.nature.com/articles/srep01882). What is the novelty in the present work? Unfortunately, the authors did not cite the article and did not mention the advancement of the work in the COF and relevant importance of the work.

7. The authors have examined how intercalation and high pressure affect the volume and unit cell dimensions of COF materials, but their analysis is incomplete. They should also address the corresponding changes in surface area and density of these designed COFs, as these properties are crucial for understanding the full scope of structural modifications and potential applications.

8. How does incorporating two different metal atoms at various positions within a single COF framework affect its properties? The study should investigate how this dual-metal intercalation influences both the electronic band gap tuning and structural characteristics of the material.

9. The authors stated that “Ca (II), having no electrons in the 4s or 3d orbitals, contributes only through lower-lying 3s and 3p states to occupied bands. As a result, the highest occupied bands are dominated by states from the COF framework, as illustrated by the projected density of states (pDOS) in Figure 2d”, but as it could be seen in Figure 2d, there is a significant contribution of d orbitals of Ca (II) rather than 3p orbitals. The spin density and oxidation states of the Ca during the intercaltion need to be explained here

10. “COF-1-Ca (0) displays one of the lowest band gaps of 0.45 eV, and Ca (II) exhibits the largest band gap at ambient pressure”. The authors should provide a more thorough

explanation addressing whether this disparity is primarily driven by the calcium's oxidation state or whether additional factors are involved.

11. How does the extent of band gap reduction in COF-1 under pressure quantitively compare to that in the cited 2D materials (e.g., perovskites, TMDs, black phosphorus)?

12. In COF-1-Ca(0), Zn(0), and Ca(II), deviations from the expected pressure trend are noted. Have the authors identified structural or electronic descriptors that predict when such deviations occur?

13. How does stacking geometry influence electronic structure compared to other variables like oxidation state or metal intercalated? Can the authors elaborate on the origin of the lateral shifts in COF-1-Ca(II)? The stability analysis is required after the intercalation.

14. How robust are the predicted electronic trends (e.g., band gap narrowing) to the choice of exchange-correlation functional? Would hybrid functionals such as HSE06 lead to similar conclusions?

15. Are the modelled intercalated structures thermodynamically stable? There is no comparison with the experiment and previous reported data. Given the exceptions observed in Zn(0) and Ca(0), can the authors propose a descriptor (e.g., metal ion size, charge transfer, orbital overlap) that predicts deviation from the electronic band gap-tressure trend?

16. Some references are not formatted properly and many important references are missing to cite them (i) https://doi.org/10.1021/acsaelm.2c01363; (ii) https://doi.org/10.1021/jacs.8b08907; (iii) https://doi.org/10.1021/acsaelm.0c00867.

Reviewer: 2

Comments to the Author

This is a very interesting article, in which the authors discussed the effects of hydrostatic pressure and metal intercalation on the electronic properties of covalent organic frameworks (COFs) through DFT calculations. Their findings that hydrostatic pressure and metal intercalation can be used to tune the band gaps of COFs will certainly be of interest to the porous materials community, in particular to researchers who are interested in the electronic properties of COFs which are relevant to a range of applications. I believe this

manuscript is suitable for publication on The Journal of Physical Chemistry Letters. There are a few things which I hope the authors can clarify/address before acceptance:

1. The authors have used the r2SCAN, a relatively new meta-GGA functional for this work. While the original authors of this functional (Ref 35) carefully benchmarked this performance of this functional on a few selected thermochemical properties, it is not clear to me whether this functional will perform equally well on electronic properties such as bank gaps and energy levels of different electronic states in comparison with the most common GGA functionals like PBE. I would suggest the authors to provide some justification on why r2SCAN was chosen for this work, and that this particular functional can provide reasonably good description to the electronic properties of the materials considered in this study in comparison with more accurate methods such as hybrid DFT.

2. For metal intercalation, the authors considered one metal per cell. I wonder what's the effect of metal loading on the electronic properties studied here. Even for the same loading, if the authors consider a bigger supercell, will the metal atoms prefer to sit closer to each other by occupying neighbouring sites, rather than distribute homogeneously in the structure? This may be more relevant to metal atoms with spin moments of which the magnetic coupling may help to stabilise metal clustering. Related to this, it would also be useful to list the metal intercalation energies, which will provide useful information on the binding strength between metal atoms and the COF host, i.e. whether intercalated metal atoms are likely to remain stable at elevated temperatures; in related 2D materials, it is known that elevated temperature may lead to higher mobility and therefore aggregation of metal atoms.

3. For the discussion on charged systems, I am unsure how relevant this would be to experimental studies, as in experiments, it is very likely that charge balancing species will be present, which may affect the electronic properties of COFs, either directly (through electronic effects) or indirectly (through affecting the layer stacking). I suggest noting this point in the discussions. From a pure computational perspective, the discussions remain interesting to me.

Several additional minor comments:

4. The authors performed Mulliken charge analysis in this study. It is well known that Mulliken charge analysis is sensitive to basis sets. Have the authors considered other charge analysis?

5. When applying high pressure, I wonder whether the author considered other layer stacking of COF-1, either with or without metal intercalation, as other types of layer stacking (see e.g. DOI: 10.1021/acs.chemmater.1c04365) may be stabilised at higher pressure.

6. The effect of pressure on the band gap of another porous material, i.e. a breathing MOF (MIL-53) was discussed in a previous computational study (DOI: 10.1021/acs.jpcc.5b04050), and it was shown that high pressure resulted in stronger overlap in electron densities between neighbouring organic linkers and therefore small band gaps, similar to what was found in the current study.

Author's Response to Peer Review Comments:

We thank both reviewers for carefully reading our manuscript and for providing useful feedback. Below we address all comments separately (in blue).

Reviewer: 1
General Comment:
The present manuscript presents a timely and well-executed computational study on the tuning of covalent organic framework (COF) electronic properties via two distinct mechanisms: high pressure and metal intercalation. The metal intercalation is not new at all and there are several studies which the authors mentioned. Moreover, the authors claimed that the band gap of pristine COF-1 decreases by ~1 eV under compression up to 10 GPa. It is really ***very very*** high pressure and it is not easily possible to generate the pressure in experiments and the COF-1 may dissemble/dissociate and break at the 10GPa.
We agree that metal intercalation in COFs has been studied previously; however, in this work we focus on its combined effect with high pressure, which to the best of our knowledge has not yet been explored. Regarding the stability of COF-1 under pressure, we refer to the high-pressure synchrotron XRD and Raman experiments reported by Sun et al. (Ref. 30 in the main text; Ref. 11 in the SI), which show that COF-1 retains crystallinity up to 12–15 GPa.
After reviewing the manuscript on band gap tuning in metal-intercalated (Ca, Cr, Zn, and Fe) COF-1 materials, I found that this preliminary investigation of electronic properties requires substantial improvement before publication. While the authors examine intercalation and pressure effects on the electronic band gap modulation, the study lacks depth and leaves several critical questions unanswered.
Publication should only be considered after the authors address the following comments in a comprehensive manner.

1. This study exclusively focused on COF-1 (with boroxine and benzene building units), limiting its scope. The authors should expand their investigation to include COFs with alternative building units, particularly triazine-based structures. A comparative analysis examining the effects of intercalation and high pressure across these different COFs would reveal which structural motifs yield the most favorable outcomes.
We have extended our study to include the triazine-based COF IITI-0 (Ref. 12 in the SI) and added a dedicated section to the supporting information (section 4), where we present and discuss the results for the pristine COF at three different pressure values. We found that the band gap decreases with increasing pressure, reaching a metallic state already at 4 GPa.

2. The reason of choosing r2SCAN functional DFT method is not mentioned anywhere. In general, HSE06-D method provides better electronic band gap than any other DFT method. It is also strange that the authors use both Gaussian and plane wave approaches. The reason is not clear at all.
The combination of the unit cell size (two COF-1 layers) and the diffusiveness of the basis set used to properly account for the interaction of the metal with the COF, prevented us from using a hybrid functional such as HSE06 together with k-point sampling, as that would be computationally too expensive. Therefore, we opted for the r^2SCAN functional (a meta-GGA functional), which has been shown to be generally accurate for geometrical, electronic, and thermochemical properties of solid-state materials and transition metal compounds (see Refs. 4, and 7-9 in the SI). Importantly, as there is no experimental data to compare with, we are interested in capturing the correct qualitative trends. To this end, and also to answer comment 14, we have performed calculations at the Γ-point for r^2SCAN, HSE06 (sampling just the Γ-point is computationally feasible in our setup) and PBE functionals. We found that the HSE06 consistently provides larger gaps, while PBE consistently lower gaps, suggesting that the qualitative trends are the same with r^2SCAN and HSE06. We refer to the reply of comment 14 for a comprehensive discussion about these new data.
To clarify our choice of functional, we have added a discussion in the SI (section 1.2), and we point the reader to it in the main manuscript when discussing the computational details.

The Gaussian and plane-wave method is an approach based on Gaussian functions as the primary basis set (hence the typical nomenclature of double-zeta, triple-zeta, and so on for the basis sets used), and a

plane wave basis for representing the electronic density and computing the Hartree term in reciprocal space.
This approach enables efficient treatment of periodic systems with localized basis functions for the orbitals and with plane waves for the electron density, combining the advantages of both types of basis sets. It is an integral part of CP2K.

3. The authors wrote that they have computed "Molecular orbitals". Is it really true? It seems that they have computed 3D band structures then why "Molecular orbitals"?
We thank the reviewer for pointing this out. In the original manuscript, we referred to the orbital at the Γ-point as a "molecular orbital," which is a purely real function, but "crystalline orbital" is more appropriate in the context of periodic systems. We have changed the name "molecular orbital" to crystalline orbital in the revised manuscript to avoid confusion.

4. Authors claimed that "all intercalated systems exhibit relatively low bulk moduli compared to other 2D materials"; to claim this point, there should be a proper literature review and references.
We have conducted a brief literature survey and added the following paragraph on bulk moduli of other 2D-layered materials:
All intercalated systems exhibit relatively low bulk moduli compared to other two-dimensional materials, such as MoS_2 (79.5 GPa),[42] black phosphorus (34 GPa),[43] and graphite (30.8 GPa experimental,[44] ~ 30 GPa theoretical[45]). 2D hybrid perovskites have comparable bulk moduli, such as for example $(BA)_2PbBr_4$ (BA=benzylammonium) (10 GPa)[46] and $BA_2MAPb_2I_7$ (12.3 GPa).

5. In Figure 1b, it is reported that the intercalated atom binds to the benzene ring. The author must justify the reason behind choosing the benzene ring for metal intercalation. Site selection could not be decided without checking the stability of the structure. I suggest that authors should calculate the binding energy of metal interaction with all the available active sites on the COF framework.
As pointed out by the referee in comment 6, Gao et al. (Ref. 20 in the paper) investigated Ca intercalation in COF-1 and found that adsorption above the benzene ring is the most stable configuration. This finding is consistent with established organometallic chemistry, such as bis(benzene)chromium, where Cr is stabilized between two aromatic rings. To support our choice, we have computed the counterpoise-corrected binding energy for COF-1-Ca(0) with Ca adsorbed between benzene rings and between boroxine rings. The benzene-ring site was found to be slightly more stable. Moreover, for Ca(II) and Fe(II) we did geometry optimizations with the ion placed at several plausible sites: between benzene rings, between boroxine rings, and—specifically for Fe^{2+}—near a framework oxygen atom, due to iron's affinity for oxygen coordination. We then compared the total energies of the relaxed structures. For both Ca^{2+} and Fe^{2+}, the adsorption site between benzene rings also remains the most favorable. We are not reporting binding energies for the cationic species, since in reality these ions originate from precursor species and must overcome a desolvation or binding energy, which we do not model. Ignoring this leads to an overestimation of the binding strength.
All these results and additional details have been included in a dedicated paragraph in the supporting information (section 2.4).

6. Gao et al. also studied the Ca-intercalated COF-1 (See; Scientific Reports, Volume 3, Article number: 1882 (Year 2013) and https://www.nature.com/articles/srep01882). What is the novelty in the present work? Unfortunately, the authors did not cite the article and did not mention the advancement of the work in the COF and relevant importance of the work.
The paper by Gao et al. (Sci. Rep. 3, 1882 (2013)) was already cited in our original submission, but we now added "Ca was specifically included as it was also part of the study of Gao *et al.*[20]" Their study involves a much higher metal loading (multiple atoms per layer and in every interlayer space) and targets hydrogen storage. In contrast, our work focuses on the effect of low metal loading and high pressure on the electronic structure, which represents a different scope and application.

7. The authors have examined how intercalation and high pressure affect the volume and unit cell

dimensions of COF materials, but their analysis is incomplete. They should also address the corresponding changes in surface area and density of these designed COFs, as these properties are crucial for understanding the full scope of structural modifications and potential applications.
We have added section 2.2 to the SI reporting the surface area, helium volume, and density of the pristine and metal-intercalated COFs at ambient pressure.

8. How does incorporating two different metal atoms at various positions within a single COF framework affect its properties? The study should investigate how this dual-metal intercalation influences both the electronic band gap tuning and structural characteristics of the material.
We agree with the reviewer that studying dual-metal intercalation would be highly interesting and could lead to additional tunability of COF properties. However, this lies beyond the scope of the present work, which focuses on isolating and understanding the individual effects of pressure and single-metal intercalation on the electronic structure.

9. The authors stated that "*Ca (II), having no electrons in the 4s or 3d orbitals, contributes only through lower-lying 3s and 3p states to occupied bands. As a result, the highest occupied bands are dominated by states from the COF framework, as illustrated by the projected density of states (pDOS) in Figure 2d*", but as it could be seen in Figure 2d, there is a significant contribution of *d* orbitals of Ca (II) rather than *3p* orbitals. The spin density and oxidation states of the Ca during the intercalation need to be explained here.
Based on our projected density of states (Figure 2d), there is no significant contribution from the Ca(II) 3d orbitals to the occupied states. We are not sure what the reviewer is referring to.

10. "*COF-1-Ca (0) displays one of the lowest band gaps of 0.45 eV, and Ca (II) exhibits the largest band gap at ambient pressure*". The authors should provide a more thorough explanation addressing whether this disparity is primarily driven by the calcium's oxidation state or whether additional factors are involved.
We ascribe this difference to be primarily driven by the oxidation state of calcium, and the fact that 3d-bands are energetically close to each other. In Ca(0) we occupy one 3d band (with some degree of hybridization with the COF, see the PDOS in Fig. 2d and the HOCO in Fig. 3), meaning that we then have a small gap to the next 3d band. In contrast, for Ca(II), the 3d manifold remains unoccupied, such that the gap from the HOCO (which is a π-conjugated state) to the 3d bands is larger.
We have added the following sentence in the main text to explain the small band gap of COF-1-Ca(0): "We ascribe this to the partially occupied 3d states, which are energetically close to each other."

11. How does the extent of band gap reduction in COF-1 under pressure quantitatively compare to that in the cited 2D materials (e.g., perovskites, TMDs, black phosphorus)?
We have added an extensive section 5 in the SI presenting the band gap reduction of other 2D materials under pressure. In the spirit of a Letter format, we included this discussion into the supporting information and refer to it briefly in the main text.

12. In COF-1-Ca(0), Zn(0), and Ca(II), deviations from the expected pressure trend are noted. Have the authors identified structural or electronic descriptors that predict when such deviations occur?
In the case of COF-1-Ca(II), the intercalation induces a significant structural reorganization: the layers shift laterally along the ab plane, become non-planar, and the metal center is no longer sandwiched between benzene rings, which instead relocates toward the side, interacting more strongly with framework oxygen atoms (see discussion in section 2.6 of the SI). In such cases of strong structural reorganization we can expect a deviation from the trends, though, their direction and magnitude are difficult to predict a priori. Concerning Ca(0) and Zn(0) we have significantly extended the discussion in the revised manuscript (see pages 12 and 14 highlighted in red).

13. How does stacking geometry influence electronic structure compared to other variables like oxidation state or metal intercalated? Can the authors elaborate on the origin of the lateral shifts in COF-1-Ca(II)? The stability analysis is required after the intercalation.

We discuss the stacking geometry
For a discussion on the stacking influence we refer to our reply to comment 5 to Reviewer 2.

In all cases, we observe slight lateral shifts, but in COF-1-Ca(II) the layer offset is more pronounced. This results from the strong interaction between Ca(II) and an oxygen atom on one layer, while sitting on top of the center of the benzene ring of the other layer.
As a Lewis acid, Ca(II) seeks electron-rich sites like oxygen, rather than interacting with the benzene π-system, leading to the observed slip.

We performed vibrational analyses for all ambient-pressure, intercalated structures discussed and found no imaginary frequencies.

14. How robust are the predicted electronic trends (e.g., band gap narrowing) to the choice of exchange-correlation functional? Would hybrid functionals such as HSE06 lead to similar conclusions?
As discussed in our reply to comment 2, it is computationally too expensive to perform HSE06 band structure calculations with the same k-point grid as we do for r^2SCAN. However, we can compare the gap at the Γ-point obtained from calculations using a 1x1x1 k-point grid, which provides a good approximation compared to the 3x3x7 calculation (see the newly added Table S2 in the SI).
Following this strategy, we computed the gaps at Γ with HSE06 and PBE, and we found that HSE06 consistently predicts larger gaps (by about 0.8 eV on average), while PBE consistently predicts smaller gaps (by about 0.4 eV on average) compared to r^2SCAN.
Most importantly, the energetic change in the Γ-point gap due to metal intercalation is similar for all three functionals, providing evidence that the qualitative trends obtained with r^2SCAN are in line with other commonly used functionals.
We have now added an extensive discussion about this in Section 1.2 of the SI, along with 3 tables (Tables S2-S4) reporting all the values of the calculations performed with HSE06 and PBE.
We refer to our reply to comment 2 for the changes in the main text.

15. Are the modelled intercalated structures thermodynamically stable? There is no comparison with the experiment and previous reported data. Given the exceptions observed in Zn(0) and Ca(0), can the authors propose a descriptor (e.g., metal ion size, charge transfer, orbital overlap) that predicts deviation from the electronic band gap-pressure trend?
We performed vibrational analyses for all ambient-pressure, intercalated structures discussed and found no imaginary frequencies.

We compare our simulated COF-1 structure, both at ambient conditions and under pressure, with available experimental data and find good agreement. However, to our knowledge, there are no experimental measurements of the band gap for COF-1, nor for any COF under pressure, and no experimental band gap for metal-intercalated COFs.

Concerning Ca(0) and Zn(0) we have significantly extended the discussion in the revised manuscript (see page 12 and 14 highlighted in red).

16. Some references are not formatted properly and many important references are missing to cite them (i) https://doi.org/10.1021/acsaelm.2c01363; (ii) https://doi.org/10.1021/jacs.8b08907; (iii) https://doi.org/10.1021/acsaelm.0c00867.
We thank the reviewer for pointing out the improperly formatted references. We included the first (ref 24) and third reference (ref 23) in our manuscript as suggested.

Reviewer: 2
Recommendation: This paper is publishable subject to minor revisions noted. Further review is not needed.
Comments: This is a very interesting article, in which the authors discussed the effects of hydrostatic pressure and metal intercalation on the electronic properties of covalent organic frameworks (COFs) through DFT calculations. Their findings that hydrostatic pressure and metal intercalation can be used to tune the band gaps of COFs will certainly be of interest to the porous materials community, in particular to researchers who are interested in the electronic properties of COFs which are relevant to a range of applications. I believe this manuscript is suitable for publication on The Journal of Physical Chemistry Letters. There are a few things which I hope the authors can clarify/address before acceptance:
1. The authors have used the r2SCAN, a relatively new meta-GGA functional for this work. While the original authors of this functional (Ref 35) carefully benchmarked this performance of this functional on a few selected thermochemical properties, it is not clear to me whether this functional will perform equally well on electronic properties such as band gaps and energy levels of different electronic states in comparison with the most common GGA functionals like PBE. I would suggest the authors to provide some justification on why r2SCAN was chosen for this work, and that this particular functional can provide reasonably good description to the electronic properties of the materials considered in this study in comparison with more accurate methods such as hybrid DFT.
We refer to the added section 1.2 in the SI and our reply to comments 2 and 14 of Reviewer 1, where we explain our rationale for the selection of r^2SCAN. We have also performed Γ-point calculations with PBE, and found that r^2SCAN is consistent with it (and with HSE06), which is in agreement with several benchmark works assessing the performance of r^2SCAN (and SCAN) for solid-state materials.

2. For metal intercalation, the authors considered one metal per cell. I wonder what's the effect of metal loading on the electronic properties studied here. Even for the same loading, if the authors consider a bigger supercell, will the metal atoms prefer to sit closer to each other by occupying neighbouring sites, rather than distribute homogeneously in the structure? This may be more relevant to metal atoms with spin moments of which the magnetic coupling may help to stabilise metal clustering. Related to this, it would also be useful to list the metal intercalation energies, which will provide useful information on the binding strength between metal atoms and the COF host, i.e. whether intercalated metal atoms are likely to remain stable at elevated temperatures; in related 2D materials, it is known that elevated temperature may lead to higher mobility and therefore aggregation of metal atoms.
This is an interesting question, though, exploring the effect of metal loading, clustering tendencies, and temperature-driven effects would require a significantly expanded set of calculations, including ab initio molecular dynamics simulations to capture thermal effects and metal aggregation. We believe this would qualify as a completely new project and is therefore beyond the scope of this work.

That said, we can address part of the question. For Ca(0), we computed the binding energy at different adsorption sites and found that adsorption above the benzene ring is the most favorable (see section 2.4 in the SI). As discussed in our reply to comment 5 of Reviewer 1, this site is also favored for Ca(II) and Fe(II).

3. For the discussion on charged systems, I am unsure how relevant this would be to experimental studies, as in experiments, it is very likely that charge balancing species will be present, which may affect the electronic properties of COFs, either directly (through electronic effects) or indirectly (through affecting the layer stacking). I suggest noting this point in the discussions. From a pure computational perspective, the discussions remain interesting to me.
We agree with the Reviewer that in experimental systems, charge-compensating species will be present, for example in the COF pores. As noted in section 1.3 in the SI, our charged calculations employ a compensating uniform background charge, which serves for keeping the unit cell overall neutral and as an approximate model for these counterions.

Several additional minor comments:
4. The authors performed Mulliken charge analysis in this study. It is well known that Mulliken charge analysis is sensitive to basis sets. Have the authors considered other charge analysis?
While we agree that Mulliken charges are sensitive to the choice of basis set, we just use them to compare qualitatively trends within the same systems. In our case, the Mulliken analysis supports the observed trend of change in the c-axis and is used strictly for qualitative interpretation, not for quantitative conclusions.
We also looked at intrinsic atomic orbital charges. However, this is currently only implemented for Γ-point calculations in CP2K, which we considered this less suitable for this study.
Finally, we tried to compute Bader charges, though, the electron density obtained with the GTH pseudopotential used in this work does not contain peaks at the atomic positions, which are necessary to perform the Bader charge analysis.

5. When applying high pressure, I wonder whether the author considered other layer stacking of COF-1, either with or without metal intercalation, as other types of layer stacking (see e.g. DOI: 10.1021/acs.chemmater.1c04365) may be stabilised at higher pressure.
We selected the offset stacking reported as the most stable in the literature (based on computational results, see Ref. 35), and performed structural optimizations using a two-layer unit cell. This allows for relaxation of the interlayer distance and layer offset, although it does not account for more complex stacking sequences such as ABC. High-pressure experimental data (Ref. 30) show no indication of a sudden structural transition or stacking rearrangement of the pristine COF-1 up to the pressures considered, supporting the validity of our chosen stacking model.

We have extended our study to include the triazine-based COF IITI-0, which is AA stacked. The corresponding data and results are discussed in section 4 of the SI.

6. The effect of pressure on the band gap of another porous material, i.e. a breathing MOF (MIL-53) was discussed in a previous computational study (DOI: 10.1021/acs.jpcc.5b04050), and it was shown that high pressure resulted in stronger overlap in electron densities between neighbouring organic linkers and therefore small band gaps, similar to what was found in the current study.
We thank the reviewer for pointing out this interesting reference. We have added a discussion of the analogies of the band gap in breathing MIL-53 in comparison to our COF to the revised manuscript (page 12 and 14 in red and reference 48).
